# Changes in annual transcriptome dynamics of a clone of Japanese cedar (*Cryptomeria japonica* D. Don) planted under different climate conditions

**DOI:** 10.1371/journal.pone.0277797

**Published:** 2023-02-16

**Authors:** Mine Nose, So Hanaoka, Eitaro Fukatsu, Manabu Kurita, Masahiro Miura, Yuichiro Hiraoka, Taiichi Iki, Osamu Chigira, Kentaro Mishima, Makoto Takahashi, Atsushi Watanabe

**Affiliations:** 1 Forest Tree Breeding Center, Forestry and Forest Products Research Institute, Forest Research and Management Organization, Hitachi, Ibaraki, Japan; 2 Hokkaido Regional Breeding Office, Forest Tree Breeding Center, Forestry and Forest Products Research Institute, Forest Research and Management Organization, Ebetsu, Hokkaido, Japan; 3 Kansai Regional Breeding Office, Forest Tree Breeding Center, Forestry and Forest Products Research Institute, Forest Research and Management Organization, Katsuta, Okayama, Japan; 4 Faculty of Agricultural Production and Management, Shizuoka Professional University of Agriculture, Iwata, Shizuoka, Japan; 5 Tohoku Regional Breeding Office, Forest Tree Breeding Center, Forestry and Forest Products Research Institute, Forest Research and Management Organization, Takizawa, Iwate, Japan; 6 Iriomote Tropical Tree Breeding Technical Garden, Forest Tree Breeding Center, Forestry and Forest Products Research Institute, Forest Research and Management Organization, Yaeyama-gun, Okinawa, Japan; 7 Department of Forest Environmental Sciences, Faculty of Agriculture, Kyushu University, Nishi-ku, Fukuoka, Japan; Austrian Federal Research Centre for Forests BFW, AUSTRIA

## Abstract

Environmental responses are critical for plant growth and survival under different climate conditions. To elucidate the underlying biological mechanisms of environmental responses in Japanese cedar (*Cryptomeria japonica* D. Don), the annual transcriptome dynamics of common clonal trees (Godai1) planted at three different climate sites (Yamagata, Ibaraki, and Kumamoto Prefectures) were analyzed using microarrays. Both principal component analysis (PCA) and hierarchical clustering of the microarray data indicated the transition to dormant transcriptome status occurred earlier and the transition to active growth status later in the colder region. Interestingly, PCA also indicated that the transcriptomes of trees grown under three different conditions were similar during the growth period (June to September), whereas the transcriptomes differed between sites during the dormant period (January to March). In between-site comparisons, analyses of the annual expression profiles of genes for sites ‘Yamagata vs. Kumamoto’, ‘Yamagata vs. Ibaraki’, and ‘Ibaraki vs. Kumamoto’ identified 1,473, 1,137, and 925 targets exhibiting significantly different expression patterns, respectively. The total of 2,505 targets that exhibited significantly different expression patterns in all three comparisons may play important roles in enabling cuttings to adapt to local environmental conditions. Partial least-squares regression analysis and Pearson correlation coefficient analysis revealed that air temperature and day length were the dominant factors controlling the expression levels of these targets. GO and Pfam enrichment analyses indicated that these targets include genes that may contribute to environmental adaptation, such as genes related to stress and abiotic stimulus responses. This study provided fundamental information regarding transcripts that may play an important role in adaptation to environmental conditions at different planting sites.

## Introduction

Based on analyses of tree-height and tree-ring data, many studies have reported that tree growth is affected by environmental conditions [1–4]. As Japanese cedar (*Cryptomeria japonica* D. Don, also known as ‘Sugi’ in Japan) is a major forestry species in Japan, accounting for 44% of artificial forests [5], and is widely planted throughout the Japanese archipelago, understanding the effects of environmental conditions on this species is very important. The relationship between environmental conditions and phenotypic traits in Japanese cedar has been examined in several studies. A positive correlation between annual ring growth and temperature during February and March was reported by Takahashi [6], and significant correlations between tree age-height and several climatic variables such as warmth, solar radiation, precipitation, and snow depth were reported by Nishizono et al. [7]. Quantitative trait locus (QTL) analysis of several phenotypic traits in three replicated common garden experiments under three different climate conditions identified an average of 53 QTLs [8]. However, only two of these QTLs affected the same traits across all three environments [8]. Moreover, one QTL associated with growth response to drought stress was identified by QTL analysis of trees growing in two contrasting environments [9]. The small number of QTLs common between regions and a QTL with a large contribution to overall climate sensitivity would indicate that Japanese cedar is highly sensitive to environmental differences. Although these previous reports suggested that environmental conditions affect the phenotypic traits of Japanese cedar, there are no published reports of studies examining the underlying biological mechanisms.

Japanese cedar grows continuously until environmental factors (temperature, day length, etc.) or internal factors (aging, etc.) cause growth to cease (indeterminate growth), whereas the degree of annual growth of many other coniferous species, such as those of the genera *Picea* and *Pinus*, is regulated endogenously (determinate growth) [10–12]. As such, environmental conditions may exert a greater influence on the timing of growth cessation in Japanese cedar than in determinate growth species. Results from studies using growth chambers indicated that a short photoperiod and low temperature suppressed height growth of Japanese cedar [13, 14]. Prior to growth cessation in autumn, Japanese cedar trees accumulate starch and break it down into sugar in all parts of seedlings (upper, middle, and lower layer shoots, and roots) to enhance cold and frost tolerance as a means of ensuring survival under harsh winter conditions, and this process also contributes energy for bud breaking and shoot growth in the following spring [15, 16]. Soluble nitrogen and total free amino acids in the lateral shoots, stem, and roots increase at the time of growth initiation, but the levels decrease beginning in March in the roots and in April in lateral shoots expanded in the previous year, and in stems [17]. Since new lateral shoots exhibit high accumulation of soluble nitrogen and total free amino acids in May, stored nitrogenous compounds may be transported to new lateral shoots for growth [17].

A few studies of evergreen coniferous species have used time-series transcriptome analyses to investigate transcripts of needles and shoots, the key perennial organs that sense changes in environmental conditions, and these analyses revealed dramatic annual dynamics [14, 18–20]. The annual transcriptome dynamics of Japanese cedar were also clearly demonstrated in our previous report [14]. Principal component analysis of microarray data demonstrated the seasonal cycle of the transcriptome and explained the seasonal phenomena of Japanese cedar. For example, the expression of growth-related genes was up-regulated during the active growth period, and the expression of genes associated with starch metabolic processes that may contribute to cold tolerance was up-regulated during the dormant period. As the respective effects of day length and temperature interact to control this annual transcriptome dynamics [14], the dynamic pattern may change under different environmental conditions, leading in turn to physiological and phenological changes that help trees adapt and survive in different planting sites.

To investigate the changes in annual transcriptome dynamics of Japanese cedar under different environmental conditions, we planted specimens of a single clone at three sites covering a wide range of environmental conditions of natural distribution and analyzed the transcriptome. In this study, microarrays were used for transcriptome analysis. Although microarrays cannot detect novel sequences and splice variants as RNA-seq can, it is a high-throughput, cost-effective, and highly sensitive technology [21] suitable for the purpose of this study. To the best of our knowledge, this is the first report of environmental responses investigated by analyzing annual transcriptome dynamics in multiple sites in a tree species. Our data identified differentially expressed genes that may be related to molecular mechanisms controlling physiological and/or phenological differences in a clone of Japanese cedar between planting sites.

## Materials and methods

### Test sites and plant materials

**Fig 1:**
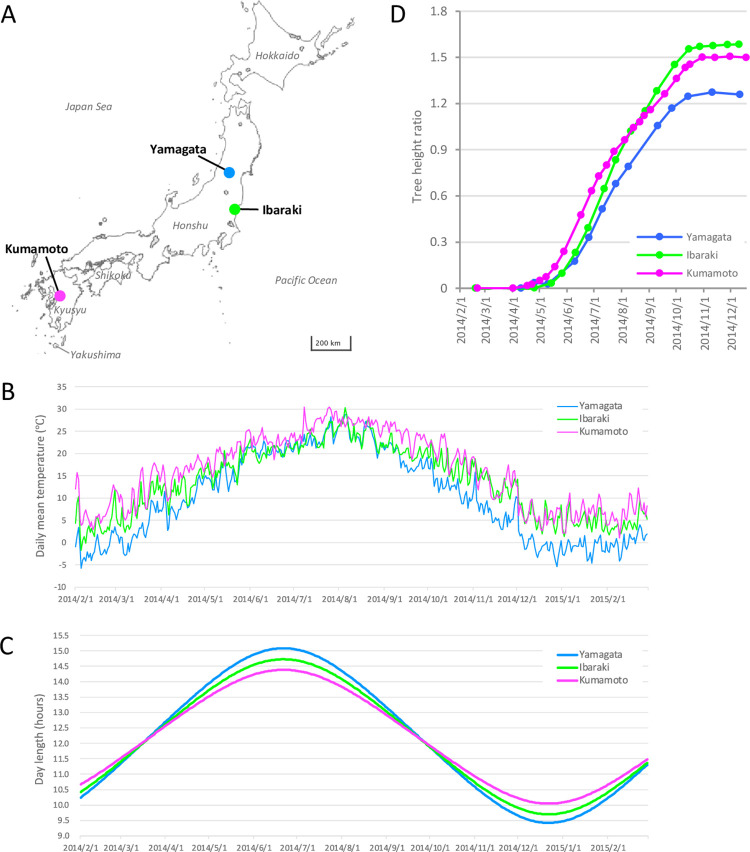
Sampling sites (A), mean air temperature (B), day length (C), and annual height growth of Japanese cedar (D) at the three study sites. We edited the map of Japan obtained from the GSI Maps Vector provided by the Geospatial Information Authority of Japan (https://maps.gsi.go.jp/vector/).

One-year-old rooted cuttings of a Japanese cedar plus-tree clone, Godai1, were planted from March to April 2013 at nurseries in three locations, Yamagata (Higashine City, Yamagata Prefecture, Japan [38°23’53"N, 140°22’47"E]), Ibaraki (Hitachi City, Ibaraki Prefecture, Japan [36°41’28"N, 140°41’21"E]), and Kumamoto (Koshi City, Kumamoto Prefecture, Japan [32°52’53"N, 130°44’5"E]) (Fig 1A). The clone ‘Godai1’ was selected from Kimitsu, Chiba (35°13’12.0"N 140°07’48.0"E) as a first-generation plus tree. We have been studying ‘Godai1’ as a model clone of Japanese cedar [14], as it shows average growth and high rooting ability for propagation [22]. A total of 10, 6, and 8 rooted cuttings were planted at more than 25-cm intervals at the respective sites. No obstacles hindered sunlight from reaching the rooted cuttings at the nurseries at the three sites. Data regarding air temperature and day length at the three sites are shown in Fig 1B and 1C. Yamagata is located in northern Japan, which is a cold region with snow cover in winter; Ibaraki is located along the coast of the Pacific Ocean and has a mild climate; and Kumamoto is located in southern Japan, which has a warm climate. Throughout the year of the study, Kumamoto exhibited the highest mean air temperature and Yamagata the lowest mean air temperature among the three sites. The mean air temperature in Ibaraki was intermediate between that of Yamagata and Kumamoto. In Ibaraki, the temperature in summer (June to September) was similar to that in Yamagata, and the temperature in winter (January to March) was similar to that in Kumamoto.

A 10-cm-long apex portion of sunny upper branches (S1 Fig) was randomly collected each month from two of the planted trees at 10:00-11:00 am from February 2014 to February 2015 (S1 Table). All sampling at the three sites was performed within 7 days. Meteorological data for the sampling days are shown in S2 Table. The height of all cuttings at the three sites was also measured at each sampling time point and other times, and growth rate was calculated by dividing the height growth differential from April 2014 by the initial height in April 2014. The significance of differences in annual growth rate (growth rate from April to December 2014) among the three sites was tested using one-way analysis of variance (ANOVA).

### RNA extraction and microarray gene expression analysis

All collected samples (3 sites × 13 time points × 2 ramets) were immediately frozen in liquid nitrogen and stored at −80°C until analysis. Total RNA was extracted from samples as reported by Gehrig *et al*. [23] using an RNeasy Plant Mini kit (Qiagen, Hilden, Germany), and DNase digestion was performed on-column using an RNase-free DNase set (Qiagen). A NanoDrop 1000 spectrophotometer (Thermo Scientific, Waltham, MA, USA) was used to accurately measure RNA concentration. RNA integrity was assessed using an Agilent 2100 bioanalyzer (Agilent Technologies, Mississauga, ON, Canada).

The microarray probes were designed using e-array (Agilent Technologies) with the default Base Composition Methodology based on isotig sequences determined from next-generation sequencing data collected from analyses of various organs (cambium region, sapwood and heartwood, tree-top, shoot, male strobili, roots of seedling) in several developmental stages and seasons using a Roche 454 Genome Sequencer [24]. A SurePrint G3 Gene Expression Custom 8×60K Array (Agilent Technologies) consisting of three probe sets corresponding to 19,304 sequences was used for microarray analyses (GenBank accession number, GPL21366) [25]. Gene annotations shown represent the top-scoring BLASTX hits for each sequence’s predicted protein product as a query against the TAIR *Arabidopsis* protein database TAIR10-pep-20101214, with a threshold e-value of 10^−5^, using the CLC Genomic Workbench software package, version 4.1.1 (CLC bio, Aarhus, Denmark).

A total of 200 μg of total RNA was transcribed to double-stranded (ds) cDNA, and the cRNA was amplified and labeled using a Low-Input Quick-Amp Labeling kit (Agilent Technologies). The cRNA was hybridized to the custom gene expression microarray for 17 h at 65°C and washed using a Gene Expression Hybridization kit (Agilent Technologies) according to the manufacturer’s instructions. The resulting slides were scanned using a SureScan Microarray Scanner G4900DA (Agilent Technologies), and scan data were compiled using Agilent Feature Extraction Software 11.5.1.1 (Agilent Technologies).

### Microarray data analysis

Raw microarray data were normalized using a 75th-percentile shift and analyzed using GeneSpring software, version 13.1 (Agilent Technologies). To enable direct comparisons of transcript profiles, median log_2_-transformed ratios for each time point were normalized to baseline (normalized intensity value). A total of 13,385 targets with a raw signal value exceeding 100 in each pair of samples under at least one of the 39 conditions (3 sites × 13 time points) were selected for further analysis. To assess the overall trend in annual transcriptome dynamics at the three sites, principal component analysis (PCA) and hierarchical clustering were performed using the 13,385 targets for the 78 samples. PCA was carried out using GeneSpring software. Hierarchical clustering was carried out using the R package “pvclust” (distance: correlation, cluster methods: average, replications: 100) [26].

To detect differences between the sites and identify statistically significant differences in profiles in the time course transcriptome data, the microarray data were analyzed using the R package ‘maSigPro’ [27]. Three between-site comparisons (‘Yamagata vs. Kumamoto’, ‘Yamagata vs. Ibaraki’, and ‘Ibaraki vs. Kumamoto’) were performed using 13 time points of normalized intensity values (cubic regression, false-discovery rate ≤10^−3^, R^2^ ≥0.7). Although a limited number of replicates from each time point were examined (2 replicates), we performed the regression analysis using a sufficient number of time series samples to detect significantly differentially expressed targets.

To identify environmental factors that affect the expression of all 2,505 of the differentially expressed targets among the three comparisons, partial least squares regression (PLSR) analysis was used. In the PLSR analysis, target expression level was used as the response variable, and the meteorological data were used as the predictor variables (S2 Table). As these predictor variables are mutually correlated, we used PLSR to account for the problem of multicollinearity. Meteorological data for the observation sites (Yamagata, observation sites in Higashine-city and Yamagata-city; Ibaraki, observation sites in Hitachi-city; and Kumamoto, observation sites in Kumamoto-city) required to calculate air temperature, precipitation, and sunlight parameters were downloaded from the Japan Meteorological Agency website (https://www.jma.go.jp/jma/indexe.html). To determine the effects of short-term and long-term temperature on the targets, seven parameters of air temperature were calculated across the sampling sites and dates, as follows: air temperature at sampling time (‘temp-0h’); minimum air temperature on the sampling day (‘temp-min’); maximum air temperature on the day before the sampling day (‘temp-max’); and mean air temperature of the previous 7, 30, 60, and 90 days (‘temp-7d’, ‘temp-30d’, ‘temp-60d’, and ‘temp-90d’, respectively). Five parameters of precipitation and five parameters of sunlight were examined: sum of previous 1, 3, 6, 24, and 72 h of precipitation (‘precip-1h’, ‘precip-3h’, ‘precip-6h’, ‘precip-24h’, and ‘precip-72h’, respectively) and sunshine duration (‘sunlight-1h’, ‘sunlight-3h’, ‘sunlight-6h’, ‘sunlight-24h’, and ‘sunlight-72h’, respectively). The day length on the sampling day at the three sites was calculated based on the time of sunrise and sunset obtained from the Ephemeris Computation Office NAOJ website (https://eco.mtk.nao.ac.jp/koyomi/index.html.en). The appropriate number of components was determined based on the explanative performance of the model (R2Y) and predictive performance of the model by cross validation (Q2Y) using the R package ‘ropls’ [28]. Correlation loading plots of the environmental parameters and the targets were created using the appropriate number of components. In addition, Pearson correlation coefficients were calculated for the normalized intensity values of the 2,505 differentially expressed targets and the meteorological data, using data from all three sites (r < −0.7, 0.7 < r, P-value < 0.001).

GO and Pfam enrichment analyses were carried out in order to determine whether the different target lists were enriched for specific GO terms or protein domains. The targets of resulting lists were categorized based on ‘GO biological process complete’ and compared to ‘*Arabidopsis thaliana* (all genes in database)’ using the PANTHER Overrepresentation Test (released 20220202; http://www.pantherdb.org/). Genes related to cold and UV response were extracted from among the differentially expressed targets according to the GO terms ‘response to cold’ and ‘response to UV’. Prior to the Pfam domain enrichment analysis, the single best ORF of each target was translated into the corresponding amino acid sequence using the tool ‘TransDecoder’, and Pfam domains of each target were identified using the tool ‘rpsblast’ [29–31]. The enrichment P-value for each Pfam domain was calculated using Fisher’s exact test in comparison to the 13,385 targets with a raw signal value exceeding 100 in each pair of samples under at least one of the 39 conditions examined (number of targets > 5, P-value < 0.01).

To further investigate the focused adaptation mechanism, genes related to hormones listed in the ‘plant hormone signal transduction’ pathways of *A*. *thaliana* in the Kyoto Encyclopedia of Genes and Genomes (KEGG; https://www.genome.jp/kegg/) and genes related to hormone biosynthesis listed in ‘hormone metabolic pathways and genes in *Arabidopsis*’ of the RIKEN Plant Hormone Research Network (http://hormones.psc.riken.jp/pathway_hormones.html) were extracted from among the 2,505 significant targets. Genes listed in the ‘starch and sucrose metabolism’ pathway in KEGG and a previous report regarding *Arabidopsis* [32] were extracted, assuming that those genes are related to starch and sugar metabolism. Similarly, genes related to amino acids were extracted according to the genes listed in the ‘biosynthesis of amino acids’ pathway in KEGG.

### Quantitative RT-PCR

To evaluate the reliability of the microarray data, five genes (peroxidase superfamily protein, lipoxygenase5, euonymus lectin S3, cytochrome P450 family 718, and glycosyl hydrolase superfamily protein) that exhibited different seasonal expression patterns in the microarray analysis were analyzed by quantitative RT-PCR (qRT-PCR) using all 78 samples. Primer pairs were designed based on next-generation sequencing isotigs using Oligo version 7.6 (Molecular Biology Insights, Inc., Vondelpark Colorado Springs, CO, USA) and Primer Express version 3.0.1 (Life Technologies) (S3 Table). The first-strand cDNA was synthesized from 500 ng of total RNA using a High Capacity RNA-to-cDNA kit (Life Technologies, Carlsbad, CA, USA). qRT-PCR was performed with Power SYBR Green PCR Master Mix (Life Technologies) and a StepOnePlus Real-time PCR system (Life Technologies), as described in the manufacturer’s instructions. Six microliters of cDNA diluted 1/24 with sterilized water was used in a reaction volume of 20 μl per well. Melting curve analysis was performed from 60 to 95°C, with data captured every 0.3°C to ensure amplification of a single product. Reaction efficiency was assessed using standard curves based on 4-fold dilution series of cDNA synthesized from 2,000 ng of total RNA (1 to 1/256 dilution). Each sample was tested independently and in triplicate using all primers. Transcript abundance was normalized to a putative *actin 7* gene, which exhibited a constant microarray value according to the Pfaffl method [33], and the data obtained for each time point were compared with data obtained for shoots collected on February 18 or July 12, 2014, in Ibaraki. Very similar expression patterns were obtained from both the microarray and qRT-PCR analyses, suggesting that the data obtained in this study were reliable (S2 Fig).

## Results

### Seasonal height growth at the three sites

At Yamagata, Ibaraki, and Kumamoto, the initial tree height (April 2014) was 59.9 cm, 50.1 cm, and 58.6 cm, respectively; the final tree height (December 2014) was 135.2 cm, 130.8 cm, and 146.7 cm, respectively; and the annual growth in 2014 was 75.3 cm, 80.8 cm, and 88.1 cm on average, respectively (S3 Fig). The height growth of the Japanese cedars began in early May in Kumamoto and late May in Yamagata and Ibaraki (Fig 1D). Growth in Ibaraki was more rapid than that in Yamagata during the growth period, from May to October. In Kumamoto, vigorous growth occurred from May to June but was slower in August and September. Height growth ceased in early October in Yamagata and Ibaraki, whereas it continued until late October in Kumamoto. ANOVA of annual growth rate indicated a significant difference in height increase among the three sites (*p* = 0.05). In Ibaraki and Kumamoto, the cuttings grew approximately 1.6- and 1.5-fold, respectively, during one growing season. The cuttings in Yamagata exhibited less height growth than those in Ibaraki and Kumamoto, with an approximately 1.3-fold growth relative to the initial height.

### Annual transcriptome dynamics and site differences

**Fig 2:**
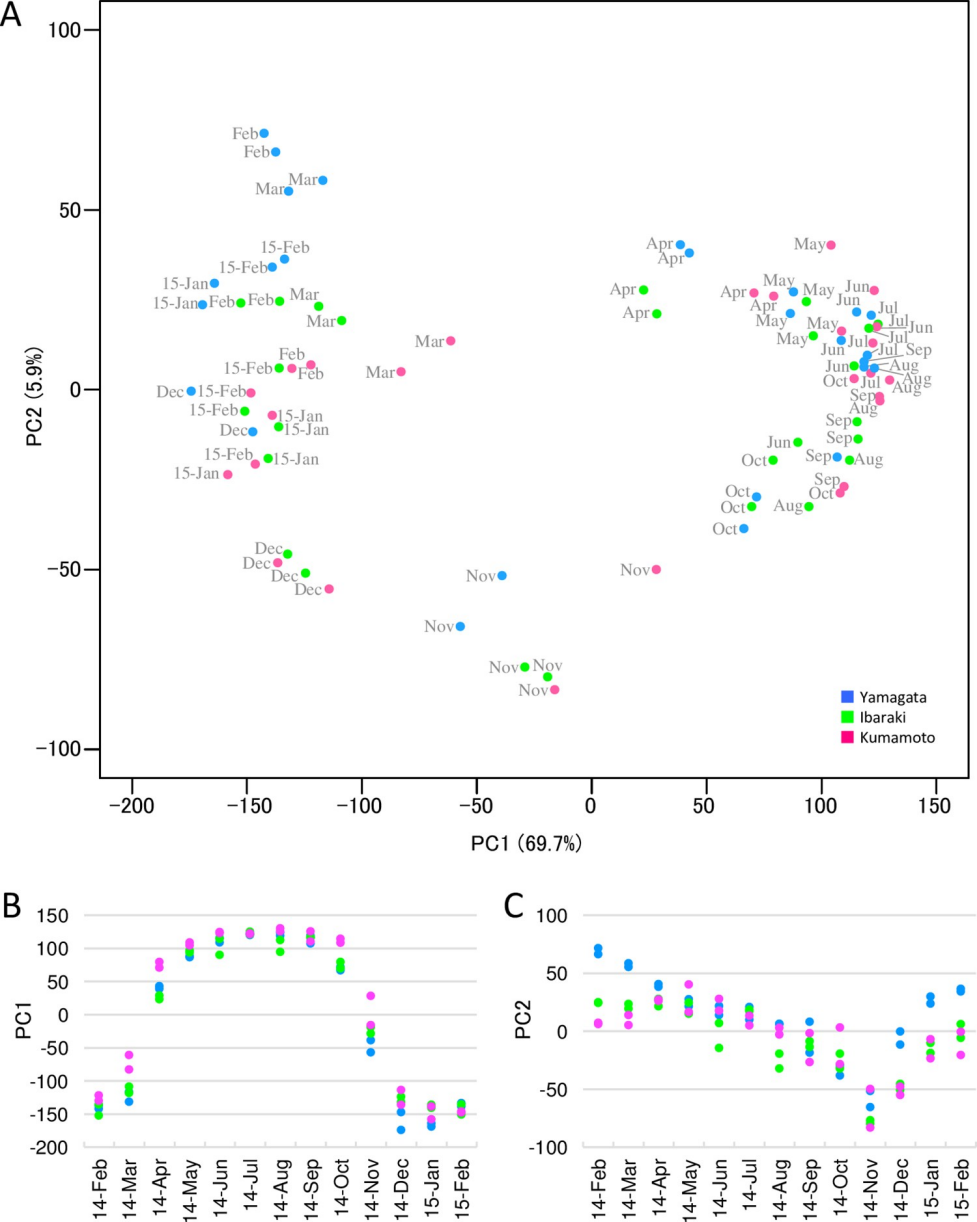
PCA of microarray data. (A) The first two principal components obtained from PCA of annual time series samples of microarray data are shown. The sample names ‘15-Jan’ and ‘15-Feb’ indicate samples collected in January and February 2015, respectively. (B) Relationship between sampling day and PC1 score. (C) Relationship between sampling day and PC2 score.

PCA was performed to obtain an overview of the annual transcriptome dynamics at the three sites (Fig 2A). The annual time series data from all sites were plotted along a circle, indicating the seasonal life cycle of Japanese cedar. PC1 explained 69.7% of the total observed variation in gene expression. The PC1 score was high in summer (from June to September) and low in winter (from December to February) (Fig 2A and 2B). PC2 explained 5.9% of the total observed variation in gene expression. The differences in PC2 may be related to the differences in transient seasons between spring (February to April) and autumn (November) (Fig 2C). Between-site variability in annual transcriptome dynamics was also revealed by PCA. The PC1 score was higher in spring (March and April) and autumn (October) at Kumamoto than at Yamagata and Ibaraki, indicating that the transcriptome remained in the growth status for longer at Kumamoto (Fig 2A and 2B). The average PC1 scores in March, April, and October at Kumamoto were 52.3, 34.4, and 54.1 points higher, respectively, than the PC1 scores at Yamagata. The PC2 score at Yamagata was higher compared with Ibaraki and Kumamoto in winter (from December to March), indicating that gene expression differed between the sites, particularly during the dormant period (Fig 2A and 2C). The difference in average PC2 score between Yamagata and Kumamoto was 48.7 during the dormant period (December to March), whereas the difference was 9.1 during the growth period (April to November).

**Fig 3:**
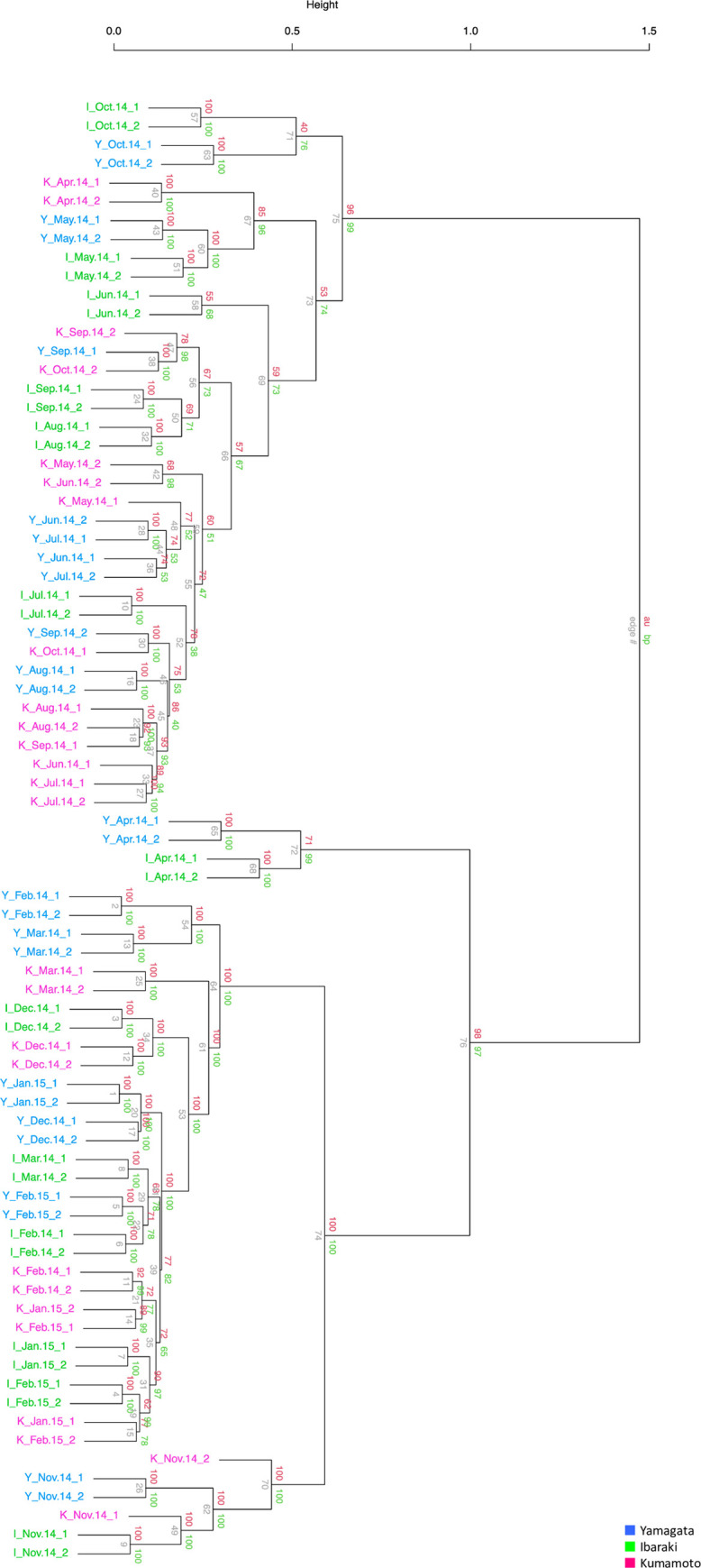
Hierarchical clustering of 78 samples collected from the three sites throughout a 1-year period.

Hierarchical clustering demonstrated two large clusters, indicating a difference between the growth period (May to October) and dormant period (November to March) (Fig 3). The samples from April divided into the two clusters; the samples from Yamagata and Ibaraki were classified in the cluster with the samples from the dormant period, and the samples from Kumamoto were classified in the cluster with the samples from the growth period. In the cluster containing growth period samples, the samples from Yamagata and Ibaraki collected in October created a sub-cluster, and the samples from Kumamoto created a cluster with other samples from the growth period. In the cluster of dormant-period samples, all six samples from November created a sub-cluster within the dormant-period cluster.

### Genes differentially expressed between sites

**Fig 4:**
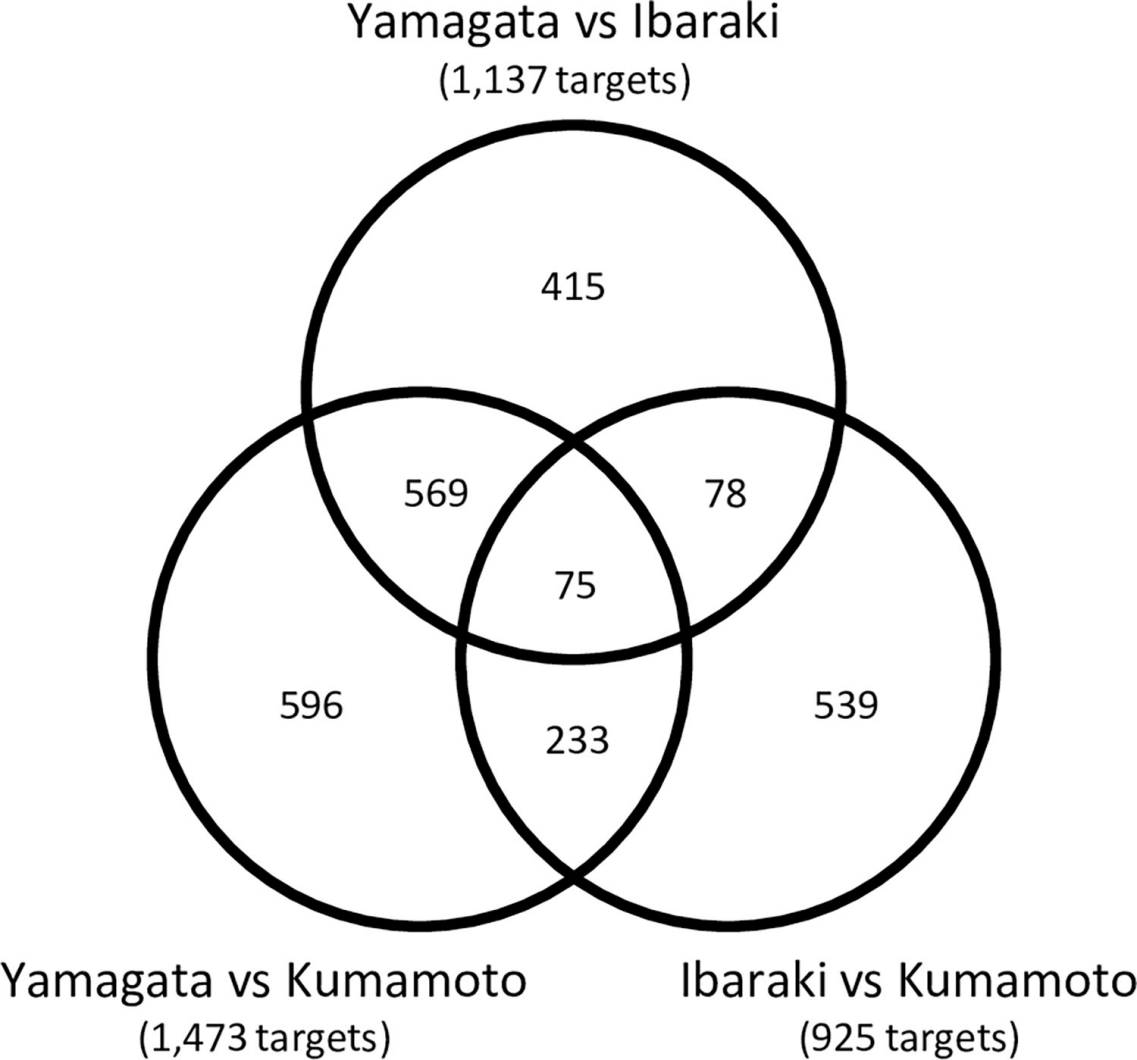
Relationship between significant differentially expressed targets from three comparisons, ‘Yamagata vs. Kumamoto’, ‘Yamagata vs. Ibaraki’, and ‘Ibaraki vs. Kumamoto’.

Analyzing annual expression patterns of the microarray data resulted in the identification of 1,473, 1,137, and 925 differentially expressed targets in the ‘Yamagata vs. Kumamoto’, ‘Yamagata vs. Ibaraki’, and ‘Ibaraki vs. Kumamoto’ comparisons, respectively (Fig 4). The comparisons revealed a total of 2,505 differentially expressed targets (S4 Table). A total of 644 differentially expressed targets were shared between the ‘Yamagata vs. Kumamoto’ and ‘Yamagata vs. Ibaraki’ comparisons, and 308 differentially expressed targets were shared between the ‘Yamagata vs. Kumamoto’ and ‘Ibaraki vs. Kumamoto’ comparisons. In contrast, only 153 differentially expressed targets were shared between the ‘Yamagata vs. Ibaraki’ and ‘Ibaraki vs. Kumamoto’ comparisons. A total of 75 differentially expressed targets were common among all three comparisons (Fig 4, Table 1).

**Table 1:** The 75 commonly significant differentially expressed targets identified in comparisons of the three sites.

Probe	Sequence ID	p-value			*Arabidopsis* ID	e-value	Symbol	Description	Pearson correlation		
		Yamagata vs Kumamoto	Ibaraki vs Kumamoto	Yamagata vs Ibaraki					parameter	r	p
CUST_14649_PI429951308	reCj29422:MSWR:isotig29274	1.42E-22	1.28E-22	1.13E-20	AT2G04520	4.4E-61	*eIF1A*	translation initiation factor eIF1A, Nucleic acid-binding, OB-fold-like protein	temp-30d	-0.95	6.10E-41
CUST_8879_PI429951308	reCj23515:MSWR:isotig23367	1.46E-22	2.89E-24	1.56E-23	AT4G22150	6.7E-104	*PUX3*	plant UBX domain-containing protein 3	temp-30d	-0.93	7.24E-35
CUST_1995_PI429951308	reCj14685:-SWR:isotig14540	8.07E-24	8.83E-19	9.60E-22	AT1G19530	2.4E-07	*-*	unknown protein	temp-max	-0.92	2.97E-32
CUST_2743_PI429951308	reCj16856:-S--:isotig16711	6.00E-16	4.52E-17	2.52E-16	AT1G52800	3.5E-39	*-*	2-oxoglutarate (2OG) and Fe(II)-dependent oxygenase superfamily protein	temp-7d	-0.90	9.39E-30
CUST_11330_PI429951308	reCj26003:MSWR:isotig25855	2.91E-22	1.72E-21	2.81E-20	AT4G30480	1.9E-73	*AtTPR1*	Tetratricopeptide repeat (TPR)-like superfamily protein	temp-0h	-0.90	1.81E-28
CUST_5940_PI429951308	reCj20543:--W-:isotig20395	2.25E-18	1.27E-22	1.42E-18	AT1G71870	0.0	*-*	MATE efflux family protein	temp-30d	-0.89	2.47E-28
CUST_11442_PI429951308	reCj26118:-SWR:isotig25970	2.56E-18	9.46E-25	1.12E-17	AT3G20970	2.1E-108	*ATNFU2*	NFU domain protein 4	temp-7d	-0.89	2.72E-27
CUST_8195_PI429951308	reCj22821:-SWR:isotig22673	4.54E-20	2.02E-21	3.35E-19	AT3G51840	0.0	*ACX4*	acyl-CoA oxidase 4	temp-0h	-0.88	1.16E-26
CUST_12080_PI429951308	reCj26768:-SWR:isotig26620	2.58E-19	5.01E-22	5.60E-17	AT3G25040	3.0E-116	*ERD2B*	endoplasmic reticulum retention defective 2B	temp-0h	-0.88	1.25E-26
CUST_1458_PI429951308	reCj13041:MSWR:isotig12896	3.37E-18	3.27E-21	5.80E-16	AT3G15010	2.0E-77	*-*	RNA-binding (RRM/RBD/RNP motifs) family protein	temp-7d	-0.88	1.85E-26
CUST_2162_PI429951308	reCj15162:-SW-:isotig15017	1.52E-17	4.46E-23	6.96E-16	AT2G03430	3.8E-102	*-*	Ankyrin repeat family protein	temp-0h	-0.88	2.92E-26
CUST_9876_PI429951308	reCj24527:MSW-:isotig24379	4.57E-16	2.78E-25	2.02E-15	AT5G61670	4.8E-135	*-*	Encodes a close homolog of the Cauliflower OR (Orange) protein	temp-7d	-0.88	3.05E-26
CUST_11060_PI429951308	reCj25730:MSWR:isotig25582	3.28E-17	1.13E-15	1.51E-15	AT2G45640	2.1E-50	*ATSAP18*	SIN3 associated polypeptide P18	temp-30d	-0.88	4.66E-26
CUST_13352_PI429951308	reCj28079:MSWR:isotig27931	5.54E-16	5.03E-22	4.25E-15	AT1G16700	1.6E-118	*AtMLO14*	Alpha-helical ferredoxin	temp-30d	-0.88	1.11E-25
CUST_2066_PI429951308	reCj14906:----:isotig14761	1.68E-20	1.05E-15	6.52E-19	AT2G42850	6.6E-105	*CYP718*	cytochrome P450, family 718	temp-30d	-0.87	5.44E-25
CUST_8368_PI429951308	reCj22997:-SWR:isotig22849	3.04E-23	3.15E-22	6.06E-19	AT5G12410	2.5E-76	*-*	THUMP domain-containing protein	temp-0h	-0.87	1.13E-24
CUST_16933_PI429951308	reCj31836:MSWR:isotig31688	5.16E-18	1.07E-23	6.12E-16	AT5G03455	2.7E-51	*CDC25*	Rhodanese/Cell cycle control phosphatase superfamily protein	temp-30d	-0.87	1.29E-24
CUST_17920_PI429951308	reCj32885:----:isotig32737	1.67E-16	1.42E-21	6.83E-15	AT1G16740	2.4E-64	*-*	Ribosomal protein L20	temp-30d	-0.85	2.44E-23
CUST_605_PI429951308	reCj09068:MSWR:isotig08925	1.72E-19	2.61E-23	1.31E-19	AT2G35490	1.5E-90	*-*	Plastid-lipid associated protein PAP / fibrillin family protein	temp-0h	-0.85	7.51E-23
CUST_8455_PI429951308	reCj23086:-SW-:isotig22938	2.36E-18	5.74E-17	4.84E-19	AT1G12570	3.0E-132	*-*	Glucose-methanol-choline (GMC) oxidoreductase family protein	temp-30d	-0.85	7.57E-23
CUST_3780_PI429951308	reCj18369:-SW-:isotig18221	2.68E-15	1.84E-16	6.62E-19	AT4G34430	7.4E-59	*ATSWI3D*	CHB3, DNA-binding family protein	temp-0h	-0.84	6.13E-22
CUST_7739_PI429951308	reCj22362:-SWR:isotig22214	3.42E-21	1.73E-15	3.73E-17	AT1G07280	7.7E-81	*-*	Tetratricopeptide repeat (TPR)-like superfamily protein	daylength	-0.84	7.08E-22
CUST_11444_PI429951308	reCj26120:----:isotig25972	6.27E-18	1.23E-16	6.09E-19	AT5G23200	3.9E-141	*-*	unknown protein	temp-30d	-0.82	2.58E-20
CUST_8766_PI429951308	reCj23401:-SWR:isotig23253	8.37E-14	1.74E-18	1.05E-13	AT2G05830	4.1E-157	*-*	NagB/RpiA/CoA transferase-like superfamily protein	temp-0h	-0.82	4.23E-20
CUST_3412_PI429951308	reCj17997:-SWR:isotig17849	1.68E-14	3.25E-13	6.27E-17	AT1G20780	2.7E-23	*PUB44*	senescence-associated E3 ubiquitin ligase 1	temp-0h	-0.81	1.36E-19
CUST_11480_PI429951308	reCj26157:MSWR:isotig26009	1.08E-14	4.27E-18	2.69E-12	AT3G51100	1.2E-48	*-*	unknown protein	temp-30d	-0.81	3.05E-19
CUST_10665_PI429951308	reCj25327:MSWR:isotig25179	9.06E-13	3.00E-17	4.19E-14	AT4G01900	4.9E-61	*PII*	GLB1 GLNB1 homolog	temp-7d	-0.81	3.90E-19
CUST_10996_PI429951308	reCj25664:-S-R:isotig25516	2.04E-16	2.63E-18	5.01E-16	AT1G18180	8.6E-113	*-*	Protein of unknown function (DUF1295)	temp-0h	-0.81	6.58E-19
CUST_13329_PI429951308	reCj28054:MSWR:isotig27906	1.80E-14	9.38E-17	3.59E-13	AT5G51040	8.4E-48	*-*	unknown protein	temp-0h	-0.80	9.04E-19
CUST_16163_PI429951308	reCj31017:--W-:isotig30869	9.45E-15	1.16E-16	1.29E-12	AT1G01260	3.6E-07	*-*	basic helix-loop-helix (bHLH) DNA-binding superfamily protein	temp-min	-0.80	3.08E-18
CUST_11485_PI429951308	reCj26162:-SWR:isotig26014	9.42E-14	2.12E-13	2.41E-12	AT5G07910	1.3E-82	*-*	Leucine-rich repeat (LRR) family protein	temp-0h	-0.79	4.27E-18
CUST_1852_PI429951308	reCj14272:-SWR:isotig14127	4.84E-13	4.32E-18	2.40E-12	AT1G71190	5.7E-107	*SAG18*	senescence associated gene 18	temp-0h	-0.79	6.02E-18
CUST_1399_PI429951308	reCj12805:MSWR:isotig12660	1.23E-16	3.10E-12	3.68E-12	AT5G19550	0.0	*AAT2*	aspartate aminotransferase 2	temp-30d	-0.78	4.46E-17
CUST_11767_PI429951308	reCj26449:MSWR:isotig26301	6.57E-14	1.10E-17	2.97E-15	-	-	*-*	Uncharacterised conserved protein (UCP012943)	temp-30d	-0.76	1.14E-15
CUST_6388_PI429951308	reCj20995:-SWR:isotig20847	8.75E-17	4.82E-14	2.61E-17	AT1G03090	0.0	*MCCA*	methylcrotonyl-CoA carboxylase alpha chain, mitochondrial / 3-methylcrotonyl-CoA carboxylase 1	temp-0h	-0.76	1.15E-15
CUST_3521_PI429951308	reCj18106:--WR:isotig17958	1.90E-15	3.21E-14	3.30E-16	AT5G45140	0.0	*NRPC2*	nuclear RNA polymerase C2	temp-0h	-0.76	1.34E-15
CUST_11766_PI429951308	reCj26448:MSWR:isotig26300	9.99E-15	5.80E-14	4.03E-14	AT1G19140	1.8E-98	*-*	molecular function unknown	temp-0h	-0.75	2.44E-15
CUST_1025_PI429951308	reCj11371:-SWR:isotig11226	3.08E-15	2.20E-13	7.40E-16	AT4G31200	2.8E-71	*-*	SWAP (Suppressor-of-White-APricot)/surp RNA-binding domain-containing protein	temp-0h	-0.73	1.89E-14
CUST_11140_PI429951308	reCj25810:-SWR:isotig25662	3.75E-12	9.70E-21	3.19E-14	AT4G22140	1.4E-95	*EBS*	PHD finger family protein / bromo-adjacent homology (BAH) domain-containing protein	temp-30d	-0.73	6.07E-14
CUST_15431_PI429951308	reCj30238:----:isotig30090	1.95E-14	3.14E-14	8.92E-16	AT5G67030	5.0E-08	*ABA1*	zeaxanthin epoxidase (ZEP)	daylength	-0.72	1.66E-13
CUST_3201_PI429951308	reCj17786:-SWR:isotig17638	5.59E-11	3.33E-13	3.02E-16	AT1G04700	4.3E-152	*-*	PB1 domain-containing protein tyrosine kinase	temp-0h	-0.71	1.99E-13
CUST_917_PI429951308	reCj10814:-SWR:isotig10669	7.16E-14	2.21E-15	5.21E-15	AT1G10170	0.0	*ATNFXL1*	NF-X-like 1	temp-0h	-0.71	4.90E-13
CUST_3631_PI429951308	reCj18217:-SWR:isotig18069	2.14E-14	3.35E-16	1.59E-14	AT1G64530	5.1E-179	*-*	Plant regulator RWP-RK family protein	temp-0h	-0.69	2.76E-12
CUST_3348_PI429951308	reCj17933:-SWR:isotig17785	5.86E-17	9.82E-15	2.62E-15	AT1G18270	0.0	*-*	ketose-bisphosphate aldolase class-II family protein	daylength	-0.67	2.09E-11
CUST_18945_PI429951308	reCj34025:----:isotig33877	2.25E-10	4.41E-13	5.53E-15	AT4G26466	3.2E-17	*LRE*	lorelei	temp-60d	0.66	4.78E-11
CUST_8012_PI429951308	reCj22636:-S-R:isotig22488	3.66E-16	3.41E-11	5.73E-17	AT4G31115	1.2E-49	*-*	Protein of unknown function (DUF1997)	temp-30d	0.70	1.40E-12
CUST_2805_PI429951308	reCj17005:----:isotig16860	4.95E-17	6.41E-13	9.59E-20	AT4G30380	1.4E-18	*EXLB2*	Barwin-related endoglucanase	temp-60d	0.73	2.72E-14
CUST_4672_PI429951308	reCj19267:MSW-:isotig19119	9.70E-15	1.31E-16	6.95E-15	AT2G40280	0.0	*-*	S-adenosyl-L-methionine-dependent methyltransferases superfamily protein	temp-90d	0.77	1.98E-16
CUST_11758_PI429951308	reCj26440:-SWR:isotig26292	2.73E-14	2.29E-18	2.67E-15	AT4G05320	1.5E-167	*UBQ10*	polyubiquitin 10	temp-0h	0.78	3.01E-17
CUST_5760_PI429951308	reCj20363:-S-R:isotig20215	1.02E-15	1.27E-21	1.36E-17	AT4G34860	0.0	*A/N-InvB*	Plant neutral invertase family protein	temp-0h	0.78	7.35E-17
CUST_12351_PI429951308	reCj27044:-SWR:isotig26896	1.44E-16	3.05E-14	2.60E-18	AT3G25120	1.1E-26	*-*	Mitochondrial import inner membrane translocase subunit Tim17/Tim22/Tim23 family protein	temp-0h	0.79	4.67E-18
CUST_821_PI429951308	reCj10352:-SWR:isotig10208	9.23E-17	5.86E-13	1.67E-14	AT1G15460	0.0	*BOR4*	HCO3- transporter family	temp-0h	0.79	1.09E-17
CUST_8015_PI429951308	reCj22639:MSWR:isotig22491	4.97E-18	6.49E-17	4.01E-17	AT3G20320	1.2E-145	*ABCI15*	TGD2 trigalactosyldiacylglycerol2	temp-30d	0.81	4.19E-19
CUST_10978_PI429951308	reCj25645:MSWR:isotig25497	6.56E-21	8.64E-20	2.77E-21	-	-	*-*	hydroxyproline-rich glycoprotein family protein	temp-30d	0.82	2.16E-20
CUST_1465_PI429951308	reCj13060:-SWR:isotig12915	2.06E-13	2.73E-15	3.88E-17	AT3G46510	3.4E-89	*PUB13*	plant U-box 13	temp-60d	0.83	2.46E-21
CUST_1000_PI429951308	reCj11257:-SWR:isotig11112	1.40E-17	6.35E-21	1.39E-18	AT5G54380	0.0	*THE1*	THE1 protein kinase family protein	temp-0h	0.83	2.68E-21
CUST_9805_PI429951308	reCj24454:-S-R:isotig24306	1.08E-14	1.00E-17	4.01E-14	AT1G80170	8.4E-146	*-*	Pectin lyase-like superfamily protein	temp-0h	0.83	6.44E-21
CUST_11193_PI429951308	reCj25864:-S--:isotig25716	1.12E-18	6.46E-18	1.61E-18	AT5G15180	7.2E-97	*-*	Peroxidase superfamily protein	temp-0h	0.84	7.74E-22
CUST_4787_PI429951308	reCj19383:-S-R:isotig19235	9.14E-16	4.54E-22	6.77E-14	AT4G18020	3.6E-49	*PRR2*	CheY-like two-component responsive regulator family protein	temp-max	0.85	2.72E-23
CUST_12966_PI429951308	reCj27679:-S-R:isotig27531	1.26E-19	2.61E-16	4.96E-20	AT1G17345	8.6E-16	*-*	SAUR-like auxin-responsive protein family	temp-30d	0.85	4.15E-23
CUST_14213_PI429951308	reCj28970:--W-:isotig28822	7.56E-14	8.64E-14	1.15E-15	AT5G38900	1.5E-72	*-*	Thioredoxin superfamily protein	temp-0h	0.85	1.23E-22
CUST_11955_PI429951308	reCj26640:-SWR:isotig26492	4.50E-17	5.15E-18	1.61E-17	AT2G28900	5.0E-46	*ATOEP16-L*	outer plastid envelope protein 16-1	temp-0h	0.85	1.90E-22
CUST_1289_PI429951308	reCj12414:-SW-:isotig12269	3.30E-22	6.42E-17	2.06E-20	AT1G29820	1.8E-180	*CSLB4*	Magnesium transporter CorA-like family protein	temp-30d	0.86	1.34E-23
CUST_4868_PI429951308	reCj19465:-SWR:isotig19317	6.97E-21	1.11E-21	1.46E-19	AT5G04060	0.0	*-*	S-adenosyl-L-methionine-dependent methyltransferases superfamily protein	temp-0h	0.87	1.92E-25
CUST_11290_PI429951308	reCj25962:MSWR:isotig25814	8.33E-16	3.45E-22	9.77E-16	AT1G78020	1.6E-19	*-*	Protein of unknown function (DUF581)	temp-0h	0.87	1.17E-24
CUST_4993_PI429951308	reCj19590:MSWR:isotig19442	1.64E-23	1.67E-23	7.91E-23	AT3G62660	1.7E-155	*GATL7*	galacturonosyltransferase-like 7	temp-0h	0.88	2.38E-26
CUST_8098_PI429951308	reCj22722:-S--:isotig22574	2.57E-22	1.10E-16	4.20E-16	AT2G34960	0.0	*CAT5*	cationic amino acid transporter 5	temp-7d	0.88	2.65E-26
CUST_8314_PI429951308	reCj22943:-SWR:isotig22795	1.09E-16	4.80E-24	2.24E-19	AT5G58600	4.6E-114	*TBL44*	Plant protein of unknown function (DUF828)	temp-30d	0.88	1.07E-25
CUST_7448_PI429951308	reCj22070:-S-R:isotig21922	7.94E-21	2.76E-13	3.00E-17	AT1G78770	0.0	*APC6*	anaphase promoting complex 6	temp-0h	0.89	1.13E-27
CUST_9022_PI429951308	reCj23659:--W-:isotig23511	3.49E-16	4.52E-22	2.19E-16	AT3G53810	6.2E-162	*-*	Concanavalin A-like lectin protein kinase family protein	temp-0h	0.89	2.24E-27
CUST_5875_PI429951308	reCj20478:-S-R:isotig20330	5.71E-18	2.21E-15	1.87E-18	AT3G15010	1.5E-47	*-*	RNA-binding (RRM/RBD/RNP motifs) family protein	temp-30d	0.90	7.96E-30
CUST_8996_PI429951308	reCj23632:-SW-:isotig23484	1.49E-22	3.31E-22	1.02E-21	AT1G07990	2.9E-10	*-*	SIT4 phosphatase-associated family protein	temp-0h	0.90	8.91E-30
CUST_5085_PI429951308	reCj19682:-SW-:isotig19534	1.23E-18	1.37E-15	4.47E-17	AT3G24560	2.9E-131	*RSY3*	Adenine nucleotide alpha hydrolases-like superfamily protein	temp-60d	0.91	4.82E-31
CUST_1622_PI429951308	reCj13527:--W-:isotig13382	2.30E-20	3.59E-19	8.70E-20	AT5G07590	4.7E-179	*-*	Transducin/WD40 repeat-like superfamily protein	temp-0h	0.92	2.06E-32
CUST_13146_PI429951308	reCj27865:-SWR:isotig27717	1.11E-25	1.19E-25	6.81E-23	AT2G38480	3.2E-23	*-*	Uncharacterised protein family (UPF0497)	temp-0h	0.94	3.40E-36

**Fig 5:**
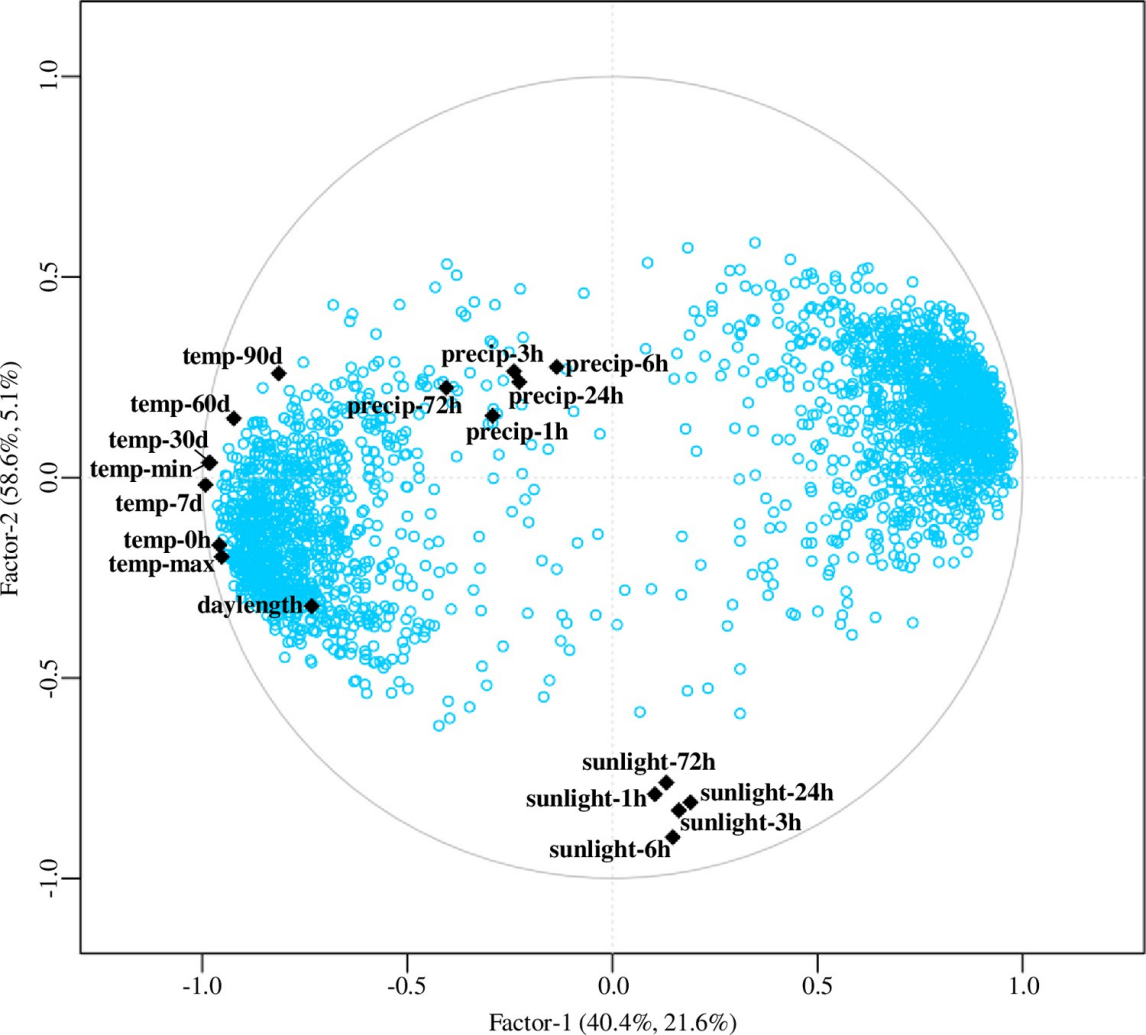
Relationship between various environmental factors and the expression of 2,505 targets exhibiting seasonal differential expression patterns between sites. Shown is a PLSR correlation-loadings plot of factor 1 versus factor 2, modeled by environmental factors (black rhombi) in the X-matrix and target expression levels (blue dots) in the Y-matrix. The first two PLS components describe 62.1% and 63.7% of the variation in the predictor variables and response variables, respectively.

The results of PLSR analyses examining the correlations between the 2,505 differentially expressed targets and meteorological parameters are shown in Fig 5. The correlations between the 2,505 targets (response variables) and latent vectors and the correlations between environmental factors (predictor variables) and latent vectors are presented. Three predictive components were used for the analyses (S5 Table), as the model had high R2Y and Q2X values (0.73 and 0.67, respectively). Parameters closed to targets were positively correlated with the nearby targets, and the meteorological parameters plotted on the opposite side (origin symmetry) were negatively correlated with the targets. As shown in Fig 5, parameters related to air temperature and day length were correlated with most of the analyzed targets. In contrast, few targets were correlated with parameters related to sunlight and precipitation.

To identify the most effective meteorological parameter for each target, correlations between the meteorological parameters and expression levels of the 2,505 differentially expressed targets were calculated (S4 Table). Most of the differentially expressed targets showed the highest correlations with air temperature and day length, with 1,842 targets (73.5%) and 222 targets (8.9%), respectively (Table 2). The parameter air temperature at sampling time (‘temp-0h’) correlated with 997 targets. The parameters of long-term air temperature more than 30 days before sampling (‘temp-30d’, ‘temp-60d’, and ‘temp-90d’) showed high correlations with 293, 104, and 35 targets, respectively. The parameters precipitation and sunlight did not show strong correlations with any of the targets. In addition, 441 targets did not show a strong correlation with any of the parameters examined in this study.

**Table 2:** Number of targets correlated with meteorological parameters.

Parameter	Number of correlated targets		Total targets
	Positive	Negative	
daylength	81	141	222
temp-0h	450	547	1842
temp-max	48	21	
temp-min	18	50	
temp-7d	94	182	
temp-30d	128	165	
temp-60d	63	41	
temp-90d	23	12	
none	-	-	441

GO enrichment analyses revealed that the 2,505 differentially expressed targets were enriched in genes associated with ‘response to stress’ and ‘response to abiotic stimulus’ (Table 3). A total of 571 targets corresponded to 485 *Arabidopsis* genes associated with ‘response to stress’, and 431 targets corresponded to 381 *Arabidopsis* genes associated with ‘response to abiotic stimulus’. Among the genes related to ‘response to stress’, 24 targets corresponding to 22 *Arabidopsis* genes associated with the GO term ‘response to cold’ exhibited a strong correlation with air temperature parameters, including *PRP31* and *F-BOX PROTEIN 7* (*FBP7*) (S6 Table). The 11 targets corresponding to 10 *Arabidopsis* genes related to the GO term ‘response to UV’ exhibited a strong negative correlation with the parameter temperature, including *ATCSA-1*, *root UVB sensitive 1* (*RUS1*), and *UV repair deficient 7* (*UVR7*) (S7 Table). Genes associated with the GO terms ‘cellular biosynthetic process’ and ‘fatty acid oxidation’ were also enriched among the 2,505 targets. Pfam enrichment analyses indicated that six domains were enriched among the 2,505 targets, including ‘Microtubule binding’ and ‘Polysaccharide biosynthesis protein’ (Table 4). All targets within the ‘Microtubule binding’ domain showed a positive correlation with air temperature (S8 Table).

**Table 3:** Functional categories overrepresented by the 2,505 targets that exhibited differential expression patterns between the three sites.

GO biological process complete	GO no.	*Arabidopsis thaliana*	Gene count	Expected	Fold enrichment	Raw P-value
organonitrogen compound metabolic process	GO:1901564	5408	531	399.24	1.33	2.77E-08
cellular macromolecule metabolic process	GO:0044260	4466	444	329.7	1.35	6.53E-07
cellular biosynthetic process	GO:0044249	2854	291	210.69	1.38	2.71E-04
cellular component assembly	GO:0022607	1052	128	77.66	1.65	8.31E-04
negative regulation of metabolic process	GO:0009892	1176	138	86.82	1.59	1.82E-03
response to stress	GO:0006950	5305	485	391.64	1.24	2.47E-03
small molecule catabolic process	GO:0044282	240	42	17.72	2.37	6.92E-03
fatty acid oxidation	GO:0019395	41	15	3.03	4.96	1.14E-02
proteolysis	GO:0006508	878	106	64.82	1.64	1.41E-02
nucleobase-containing compound metabolic process	GO:0006139	2203	224	162.63	1.38	1.43E-02
phenylpropanoid metabolic process	GO:0009698	118	26	8.71	2.98	1.59E-02
monocarboxylic acid catabolic process	GO:0072329	63	18	4.65	3.87	2.31E-02
response to chemical	GO:0042221	5130	461	378.72	1.22	3.14E-02
lipid biosynthetic process	GO:0008610	762	93	56.25	1.65	4.11E-02
response to abiotic stimulus	GO:0009628	4145	381	306	1.25	4.15E-02

The targets were compared with *Arabidopsis* genes to identify overrepresented categories (false-discovery rate ≤0.05). Only the lowest categories in the GO hierarchy are listed.

**Table 4:** Enrichment analysis of Pfam protein domains among the 2,505 targets that exhibited differential expression patterns between the three sites.

Pfam no.	Name	Reference	Count	Fold enrichment	P-value
pfam00248	Aldo/keto reductase family	33	15	2.26	0.011
pfam12146	Hydrolase 4, Serine aminopeptidase, S33	41	17	2.06	0.020
pfam00561	Abhydrolase 1, alpha/beta hydrolase fold	70	24	1.71	0.027
pfam01370	Epimerase, 0D dependent epimerase/dehydratase family	78	25	1.59	0.047
pfam02719	Polysacc synt 2, Polysaccharide biosynthesis protein	20	9	2.24	0.047
pfam16796	Microtubule binding	20	9	2.24	0.047

The 824 targets positively correlated with the parameters of air temperature were enriched in genes associated with ‘microtubule cytoskeleton organization’, ‘plant-type cell wall biogenesis’, ‘developmental growth’, and ‘cell division’, which may be related to organization and growth (Table 5). In contrast, 1,018 targets negatively correlated with air temperature were enriched in genes associated with ‘organic substance metabolic process’, ‘fatty acid beta-oxidation’, and ‘gene expression’. No statistically significant results were obtained for targets correlated with day length.

**Table 5:** Functional categories overrepresented among targets correlated with the parameters of air temperature.

GO biological process complete		GO no.	*Arabidopsis thaliana*	Gene count	Expected	Fold enrichment	Raw P-value
positive correlation to air temperature (824 targets)			* *				
	organic substance metabolic process	GO:0071704	9787	321	246.55	1.3	3.19E-05
	cellular metabolic process	GO:0044237	9520	309	239.82	1.29	2.67E-04
	microtubule cytoskeleton organization	GO:0000226	166	17	4.18	4.07	9.24E-03
	plant-type cell wall biogenesis	GO:0009832	271	22	6.83	3.22	1.23E-02
	primary metabolic process	GO:0044238	8104	260	204.15	1.27	2.28E-02
	developmental growth	GO:0048589	1101	53	27.74	1.91	4.52E-02
	cell division	GO:0051301	483	30	12.17	2.47	4.67E-02
negative correlation to air temperature (1,018 targets)							
	organic substance metabolic process	GO:0071704	9787	444	315.05	1.41	9.31E-15
	fatty acid beta-oxidation	GO:0006635	37	12	1.19	10.07	7.57E-05
	gene expression	GO:0010467	1686	95	54.27	1.75	9.47E-04
	ribonucleoprotein complex biogenesis	GO:0022613	442	37	14.23	2.6	2.1E-03
	establishment of protein localization to organelle	GO:0072594	272	26	8.76	2.97	8.2E-03
	organic cyclic compound metabolic process	GO:1901360	3066	146	98.7	1.48	9.7E-03
	cellular biosynthetic process	GO:0044249	2854	137	91.87	1.49	1.39E-02
	response to chemical	GO:0042221	5130	221	165.14	1.34	1.59E-02
	iron-sulfur cluster assembly	GO:0016226	36	9	1.16	7.77	2.61E-02
	cellular nitrogen compound metabolic process	GO:0034641	3186	148	102.56	1.44	2.98E-02
	primary alcohol metabolic process	GO:0034308	19	7	0.61	11.44	3.31E-02
	ubiquitin-dependent protein catabolic process	GO:0006511	550	39	17.71	2.2	3.43E-02
	small molecule biosynthetic process	GO:0044283	913	56	29.39	1.91	3.54E-02
	cellular aromatic compound metabolic process	GO:0006725	2957	138	95.19	1.45	4.29E-02

The targets were compared with *Arabidopsis* genes to identify overrepresented categories (false-discovery rate ≤0.05). Only the lowest categories in the GO hierarchy are listed. There were no overrepresented categories for targets correlated with day length.

We extracted 40 plant hormone–related gene targets from the 2,505 differentially expressed targets (Table 6). Five targets were related to abscisic acid (ABA); three of these targets were negatively correlated with ‘temp-0h’, and two of them were negatively correlated with ‘day length’. Four targets were related to ethylene, and all four showed a strong negative correlation with short-term temperature (‘temp-0h’ and ‘temp-max’). Five, six, three, and seven targets were related to growth-related hormones, brassinosteroid, cytokinin, gibberellin, and auxin, respectively. Two brassinosteroid-related genes (brassinosteroid 23-O-glucosilase [*UGT73C5*], and putative brassinosteroid hydroxylase [*CPD/CYP90A1*]), two cytokinin-related genes (adenine phosphoribosyl transferase [*APT3*], and response regulator 1 [*RR1*]), and four auxin-related genes (tryptophan synthase beta subunit [*TSB2*], SAUR family protein, transport inhibitor response 1 [*TIR1*], tryptophan synthase beta subunit homolog [*TSB2 homolog*]) showed a strong negative correlation with the parameters of air temperature.

**Table 6:** Hormone-related genes that exhibited differential expression patterns among the three sites.

Hormone	Probe	Sequence ID	*Arabidopsis* ID	e-value	Symbol	Description	Parameter	Pearson r
ABA	CUST_10650_PI429951308	reCj25311:--WR:isotig25163	AT2G29380	2.1E-94	*HAI3*	highly ABA-induced PP2C protein 3	temp-0h	-0.91
	CUST_4971_PI429951308	reCj19568:-SWR:isotig19420	AT3G56850	5.0E-51	*AREB3*	ABA-responsive element binding protein 3	temp-0h	-0.89
	CUST_4638_PI429951308	reCj19233:MSW-:isotig19085	AT1G49720	2.5E-38	*ABF1*	abscisic acid responsive element-binding factor 1	daylength	-0.85
	CUST_11959_PI429951308	reCj26644:--W-:isotig26496	AT2G29380	1.2E-74	*HAI3*	highly ABA-induced PP2C protein 3	temp-0h	-0.83
	CUST_15431_PI429951308	reCj30238:----:isotig30090	AT5G67030	5.0E-08	*ABA1/ZEP*	zeaxanthin epoxidase	daylength	-0.72
ACC	CUST_4315_PI429951308	reCj18908:MSWR:isotig18760	AT2G25490	2.5E-151	*EBF1*	EIN3-binding F-box protein 1	temp-0h	-0.86
	CUST_3224_PI429951308	reCj17809:-SWR:isotig17661	AT5G03280	2.9E-107	*EIN2*	ethylene-insensitive protein 2	temp-0h	-0.81
	CUST_4277_PI429951308	reCj18869:-SW-:isotig18721	AT3G23150	0.0	*ETR2*	ethylene receptor 2	temp-max	-0.78
	CUST_3849_PI429951308	reCj18439:MSWR:isotig18291	AT2G27050	1.7E-167	*EIL1*	ethylene insensitive 3-like 1 protein	temp-0h	-0.78
BR	CUST_8680_PI429951308	reCj23313:MS--:isotig23165	AT2G36800	2.2E-64	*UGT73C5*	brassinosteroid 23-O-glucosilase	temp-0h	-0.84
	CUST_13198_PI429951308	reCj27919:----:isotig27771	AT5G05690	1.8E-41	*CPD/CYP90A1*	putative brassinosteroid hydroxylase	temp-7d	-0.84
	CUST_9341_PI429951308	reCj23981:-S--:isotig23833	AT3G50660	0.0	*DWF4/CYP90B1*	steroid C-22 hydroxylase	temp-0h	0.85
	CUST_2024_PI429951308	reCj14774:-S--:isotig14629	AT2G26710	3.0E-106	*BAS1/CYP734A1*	brassinosteroid C-26 hydroxylase	temp-0h	0.87
	CUST_6644_PI429951308	reCj21252:MSWR:isotig21104	AT3G19820	0.0	*DWF1/DIM*	sterol C-24 reductase	daylength	0.90
CK	CUST_10472_PI429951308	reCj25133:MSWR:isotig24985	AT4G22570	4.8E-89	*APT3*	adenine phosphoribosyl transferase	temp-7d	-0.95
	CUST_3548_PI429951308	reCj18133:-SWR:isotig17985	AT3G16857	3.9E-117	*RR1*	response regulator 1	temp-0h	-0.85
	CUST_3656_PI429951308	reCj18243:-SWR:isotig18095	AT4G16110	1.1E-97	*RR2*	transcription factor response regulator 2	temp-0h	-0.68
	CUST_4286_PI429951308	reCj18878:-SW-:isotig18730	AT4G16110	6.7E-117	*RR2*	transcription factor response regulator 2	temp-90d	-0.63
	CUST_15877_PI429951308	reCj30715:---R:isotig30567	AT2G28305	1.8E-121	*AtLOG1*	cytokinin nucleoside 5’-monophosphate phophoribohydrolase	temp-0h	0.69
	CUST_12266_PI429951308	reCj26958:-SW-:isotig26810	AT1G27450	5.6E-83	*APT1*	adenine phosphoribosyl transferase	temp-0h	0.85
GA	CUST_13090_PI429951308	reCj27806:--W-:isotig27658	AT4G21200	3.7E-71	*AtGA2ox8*	GA 2-oxidase (class III)	daylength	0.59
	CUST_10514_PI429951308	reCj25175:-S--:isotig25027	AT5G56300	1.6E-135	*GAMT2*	GA methyltransferase	daylength	0.73
	CUST_4345_PI429951308	reCj18938:-S--:isotig18790	AT4G02780	0.0	*AtCPS/GA1*	ent-copalyl diphosphate synthase	temp-7d	0.87
IAA	CUST_7931_PI429951308	reCj22555:MSWR:isotig22407	AT4G27070	0.0	*TSB2/TRP2*	tryptophan synthase beta subunit	temp-7d	-0.91
	CUST_13907_PI429951308	reCj28650:-SW-:isotig28502	AT3G43120	1.3E-27	*-*	K14488 SAUR family protein	temp-0h	-0.87
	CUST_818_PI429951308	reCj10307:MSW-:isotig10163	AT3G62980	2.4E-140	*TIR1*	transport inhibitor response 1	temp-30d	-0.85
	CUST_9836_PI429951308	reCj24485:--W-:isotig24337	AT5G38530	0.0	*TSB2 homolog*	tryptophan synthase beta subunit homolog	temp-0h	-0.82
	CUST_13898_PI429951308	reCj28641:M-WR:isotig28493	AT1G04100	2.3E-24	*IAA10*	auxin-responsive protein IAA10	daylength	-0.51
	CUST_12991_PI429951308	reCj27704:-SWR:isotig27556	AT4G14550	9.5E-76	*IAA14*	auxin-responsive protein IAA14	temp-30d	0.69
	CUST_16743_PI429951308	reCj31635:----:isotig31487	AT2G21220	7.5E-30	*-*	K14488 SAUR family protein	temp-0h	0.85
JA	CUST_8195_PI429951308	reCj22821:-SWR:isotig22673	AT3G51840	0.0	*ACX4*	acyl-CoA oxidase	temp-0h	-0.88
	CUST_1443_PI429951308	reCj12982:MSW-:isotig12837	AT1G06290	0.0	*ACX3*	acyl-CoA oxidase	daylength	-0.69
	CUST_9900_PI429951308	reCj24551:-S-R:isotig24403	AT5G42650	9.1E-101	*AOS*	allene oxide synthase (CYP74A1)	temp-90d	-0.48
	CUST_12865_PI429951308	reCj27575:-S--:isotig27427	AT1G20510	2.9E-172	*OPCL1*	OPC-8:0-CoA ligase	temp-90d	0.40
	CUST_9193_PI429951308	reCj23831:-S--:isotig23683	AT2G46370	4.2E-168	*JAR1*	jasmonic acid-amido synthetase JAR1	temp-90d	0.81
	CUST_1324_PI429951308	reCj12520:MS-R:isotig12375	AT5G20900	2.8E-16	*JAZ12*	protein TIFY 3B	temp-0h	0.86
SA	CUST_2395_PI429951308	reCj15941:---R:isotig15796	AT2G23620	4.1E-56	*AtMES1*	methyl salicylate esterase	temp-max	-0.91
	CUST_16310_PI429951308	reCj31173:---R:isotig31025	AT4G33720	5.9E-62	*-*	K13449 pathogenesis-related protein 1	temp-90d	-0.70
	CUST_4723_PI429951308	reCj19318:-SWR:isotig19170	AT5G45110	1.7E-133	*NPR3*	NPR1-like protein 3	temp-0h	-0.68
	CUST_17831_PI429951308	reCj32790:----:isotig32642	AT4G33720	8.2E-62	*-*	K13449 pathogenesis-related protein 1	temp-0h	0.93

Hormones are abbreviated as follows: ABA, abscisic acid; ACC, 1-aminocyclopropane-1-carboxylic acid (ethylene); BR, brassinosteroid; CK, cytokinin; GA, gibberellin; IAA, indole-3-acetic acid (auxin); JA, jasmonic acid; SA, salicylic acid.

Twenty-three targets corresponding to 20 starch- and sugar-related genes were extracted from among the 2,505 differentially expressed targets (Table 7), and these targets showed variations in expression. Expression levels of the starch synthesis–related genes phosphoglucomutase (*PGM*), AGPase large subunit 2 (*APL2*), and soluble starch synthase 2 (*SS2*) were strongly positively correlated with the air temperature parameters ‘temp-0h’ and ‘temp-30d’. Expression levels of the starch degradation–related genes like sex four 1 (*LSF1*) and beta-amylase 4 (*BAM4*) were negatively correlated with ‘temp-0h’, and expression levels of the gene glucan water dikinase 1 (*GWD1*) were negatively correlated with ‘day length’.

**Table 7:** Starch- and sugar-related genes exhibiting differential expression patterns among the three sites.

Probe	Sequence ID	*Arabidopsis* ID	e-value	Symbol	Description	Parameter	Pearson r
CUST_8654_PI429951308	reCj23286:MSWR:isotig23138	AT1G66430	1.1E-179	*-*	pfkB-like carbohydrate kinase family protein	temp-30d	-0.88
CUST_6435_PI429951308	reCj21042:MSWR:isotig20894	AT1G70730	0.0	*PGM2*	phosphoglucomutase/phosphomannomutase family protein	temp-7d	-0.85
CUST_5677_PI429951308	reCj20278:MSWR:isotig20130	AT3G01510	0.0	*LSF1*	like sex four 1	temp-0h	-0.84
CUST_3410_PI429951308	reCj17995:MSWR:isotig17847	AT4G17770	0.0	*TPS5*	trehalose phosphatase/synthase 5	temp-0h	-0.83
CUST_3679_PI429951308	reCj18266:-SWR:isotig18118	AT1G68020	0.0	*ATTPS6*	UDP-Glycosyltransferase / trehalose-phosphatase family protein	temp-7d	-0.78
CUST_3400_PI429951308	reCj17985:-SWR:isotig17837	AT1G10760	0.0	*SEX1/GWD1*	glucan, water dikinase 1	daylength	-0.75
CUST_7078_PI429951308	reCj21692:MSWR:isotig21544	AT5G55700	0.0	*BAM4*	beta-amylase 4	temp-0h	-0.73
CUST_3989_PI429951308	reCj18579:-SWR:isotig18431	AT1G69830	3.3E-165	*AMY3*	alpha-amylase-like 3	daylength	-0.69
CUST_8535_PI429951308	reCj23167:---R:isotig23019	AT4G29680	0.0	*-*	alkaline-phosphatase-like family protein	temp-60d	-0.68
CUST_5435_PI429951308	reCj20035:MSWR:isotig19887	AT4G29130	0.0	*HXK1*	hexokinase 1	temp-90d	-0.66
CUST_12734_PI429951308	reCj27439:-S--:isotig27291	AT2G01630	6.2E-83	*-*	O-glycosyl hydrolases family 17 protein	temp-90d	-0.59
CUST_3803_PI429951308	reCj18392:-SW-:isotig18244	AT1G03310	0.0	*ATBE2*	Isoamylase 2	daylength	-0.46
CUST_3868_PI429951308	reCj18458:-SWR:isotig18310	AT3G46970	0.0	*ATPHS2*	a-glucan phosphorylase 2	temp-90d	0.67
CUST_18467_PI429951308	reCj33484:MS--:isotig33336	AT4G22100	1.7E-42	*BGLU3*	beta glucosidase 2	temp-90d	0.68
CUST_7046_PI429951308	reCj21660:-SWR:isotig21512	AT2G01630	0.0	*-*	O-glycosyl hydrolases family 17 protein	daylength	0.79
CUST_11925_PI429951308	reCj26609:----:isotig26461	AT3G01180	1.8E-67	*SS2*	starch synthase 2	temp-0h	0.82
CUST_8280_PI429951308	reCj22907:MS--:isotig22759	AT1G70710	0.0	*GH9B1*	glycosyl hydrolase 9B1	temp-0h	0.88
CUST_4959_PI429951308	reCj19556:MSWR:isotig19408	AT5G49720	0.0	*GH9A1*	glycosyl hydrolase 9A1	temp-0h	0.89
CUST_7451_PI429951308	reCj22073:MSWR:isotig21925	AT1G66430	2.4E-164	*-*	pfkB-like carbohydrate kinase family protein	temp-0h	0.89
CUST_5821_PI429951308	reCj20424:-SWR:isotig20276	AT4G24040	0.0	*TRE1*	trehalase 1	temp-30d	0.91
CUST_7431_PI429951308	reCj22053:-SW-:isotig21905	AT1G27680	0.0	*APL2*	AGPase large subunit 2	temp-0h	0.93
CUST_4967_PI429951308	reCj19564:MSWR:isotig19416	AT5G51820	0.0	*PGM*	phosphoglucomutase	temp-30d	0.93
CUST_6822_PI429951308	reCj21433:-SW-:isotig21285	AT2G01630	0.0	*-*	O-glycosyl hydrolases family 17 protein	temp-0h	0.94

**Fig 6:**
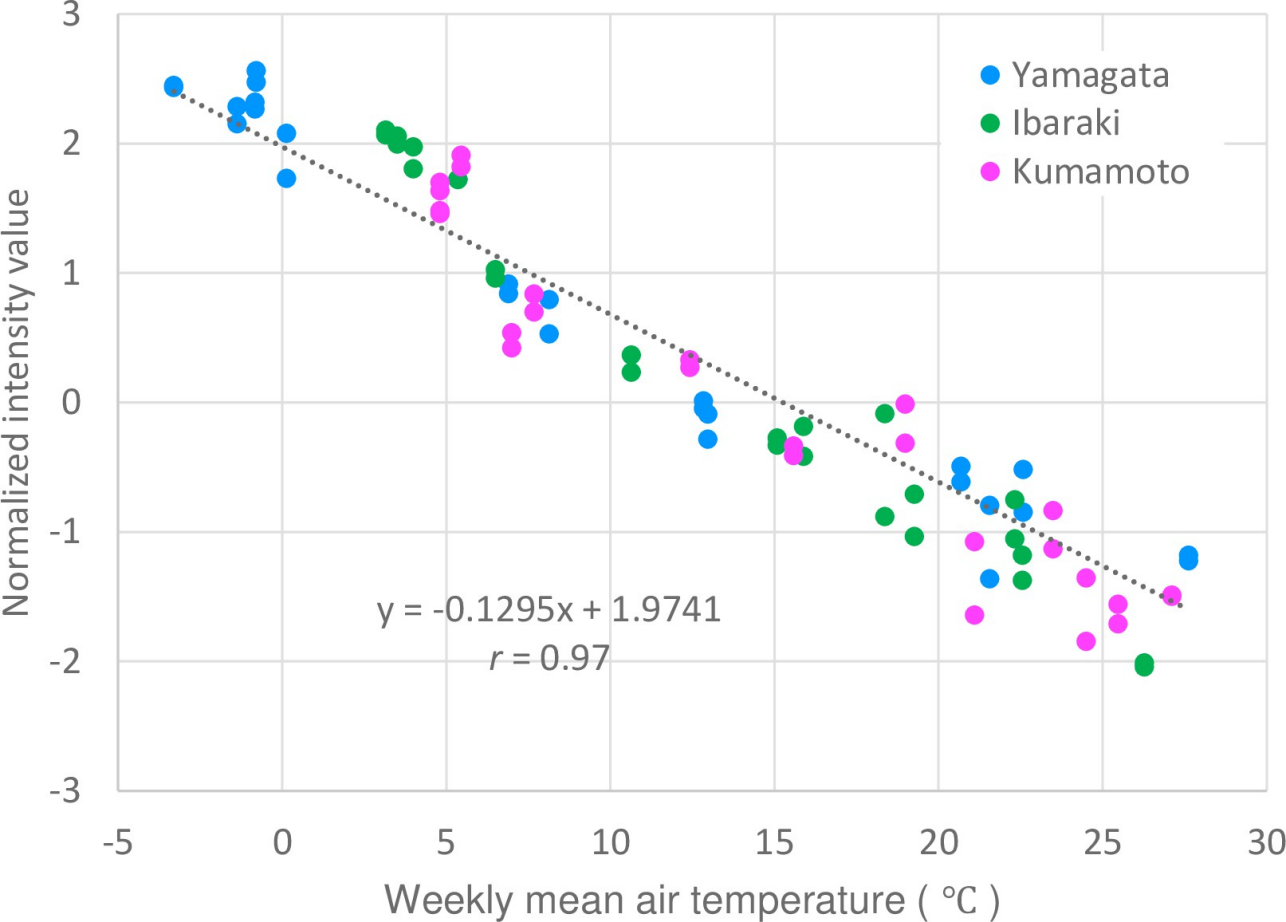
Correlation between 7-day mean air temperature and expression level of the gene encoding peptidase M20/M25/M40 family protein.

Thirty-one targets corresponding to 28 amino acid–related genes were extracted from among the 2,505 differentially expressed targets (Table 8). Of these 31 targets, 19 exhibited a strong correlation with the parameters of air temperature, such as glutamate synthesis–related genes (alanine aminotransferase 2 [*ALAAT2*] and aspartate aminotransferase 2 [*ASP2*]), citrulline synthesis–related genes (ornithine carbamoyltransferase [*OTC*], and peptidase M20/M25/M40 family protein), and a proline synthesis–related gene (pyrroline-5-carboxylate reductase [*P5CR*]). Among the 31 targets, the gene encoding peptidase M20/M25/M40 family protein exhibited the highest correlation with ‘temp-7d’ (*r* = −0.97, Fig 6).

**Table 8:** Amino acid–related genes exhibiting differential expression patterns among the three sites.

Probe	Sequence ID	*Arabidopsis* ID	e-value	Symbol	Description	Parameter	Pearson r
CUST_8719_PI429951308	reCj23354:MSWR:isotig23206	AT4G17830	0.0	*-*	Peptidase M20/M25/M40 family protein	temp-7d	-0.97
CUST_12145_PI429951308	reCj26834:--WR:isotig26686	AT5G14800	4.0E-113	*P5CR*	pyrroline-5- carboxylate (P5C) reductase	temp-7d	-0.96
CUST_10209_PI429951308	reCj24864:MSW-:isotig24716	AT3G04790	9.5E-47	*EMB3119*	Ribose 5-phosphate isomerase, type A pro	temp-7d	-0.94
CUST_7746_PI429951308	reCj22369:--W-:isotig22221	AT4G13430	0.0	*IIL1*	isopropyl malate isomerase large subunit 1	temp-0h	-0.94
CUST_8325_PI429951308	reCj22954:MSWR:isotig22806	AT4G14880	2.3E-168	*OASA1*	O-acetylserine (thiol) lyase (OAS-TL) isof	temp-7d	-0.94
CUST_5588_PI429951308	reCj20189:MSWR:isotig20041	AT1G72330	0.0	*ALAAT2*	alanine aminotransferase 2	temp-30d	-0.93
CUST_6122_PI429951308	reCj20725:MSWR:isotig20577	AT3G52990	0.0	*-*	Pyruvate kinase family protein	temp-0h	-0.91
CUST_7931_PI429951308	reCj22555:MSWR:isotig22407	AT4G27070	0.0	*TSB2*	tryptophan synthase beta-subunit 2	temp-7d	-0.91
CUST_5816_PI429951308	reCj20419:MSWR:isotig20271	AT5G52920	0.0	*PKP-BETA1*	plastidic pyruvate kinase beta subunit 1	temp-0h	-0.90
CUST_6317_PI429951308	reCj20922:-SWR:isotig20774	AT1G80600	0.0	*WIN1*	HOPW1-1-interacting 1	temp-min	-0.89
CUST_5166_PI429951308	reCj19763:MSWR:isotig19615	AT3G10050	0.0	*OMR1*	L-O-methylthreonine resistant 1	temp-7d	-0.87
CUST_7163_PI429951308	reCj21779:MSWR:isotig21631	AT4G13430	0.0	*IIL1*	isopropyl malate isomerase large subunit 1	temp-7d	-0.86
CUST_11543_PI429951308	reCj26221:MSW-:isotig26073	AT3G01850	6.6E-131	*-*	Aldolase-type TIM barrel family protein	temp-max	-0.84
CUST_9836_PI429951308	reCj24485:--W-:isotig24337	AT5G38530	0.0	*TSBtype2*	tryptophan synthase beta type 2	temp-0h	-0.82
CUST_1398_PI429951308	reCj12804:M---:isotig12659	AT5G19550	0.0	*ASP2*	aspartate aminotransferase 2	temp-30d	-0.81
CUST_8860_PI429951308	reCj23496:MSWR:isotig23348	AT1G75330	8.7E-174	*OTC*	ornithine carbamoyltransferase	temp-30d	-0.80
CUST_6322_PI429951308	reCj20927:MSWR:isotig20779	AT3G52990	0.0	*-*	Pyruvate kinase family protein	temp-60d	-0.79
CUST_1399_PI429951308	reCj12805:MSWR:isotig12660	AT5G19550	0.0	*ASP2*	aspartate aminotransferase 2	temp-30d	-0.78
CUST_6383_PI429951308	reCj20989:MSWR:isotig20841	AT3G57050	0.0	*CBL*	cystathionine beta-lyase	temp-0h	-0.78
CUST_4351_PI429951308	reCj18944:-SWR:isotig18796	AT3G22960	0.0	*PKP-ALPHA*	Pyruvate kinase family protein	daylength	-0.77
CUST_12927_PI429951308	reCj27637:---R:isotig27489	AT3G07630	4.7E-66	*ADT2*	arogenate dehydratase 2	temp-30d	-0.65
CUST_4726_PI429951308	reCj19321:MSWR:isotig19173	AT3G55610	0.0	*P5CS2*	delta 1-pyrroline-5-carboxylate synthase 2	temp-90d	-0.58
CUST_9575_PI429951308	reCj24221:MSWR:isotig24073	AT3G17390	0.0	*MTO3*	S-adenosylmethionine synthetase family prot	temp-0h	0.56
CUST_8892_PI429951308	reCj23528:MSWR:isotig23380	AT2G45440	0.0	*DHDPS2*	dihydrodipicolinate synthase	temp-0h	0.71
CUST_10056_PI429951308	reCj24708:----:isotig24560	AT3G12780	2.1E-50	*PGK1*	phosphoglycerate kinase 1	temp-60d	0.73
CUST_1587_PI429951308	reCj13430:----:isotig13285	AT4G24830	0.0	*-*	arginosuccinate synthase family	temp-0h	0.75
CUST_1081_PI429951308	reCj11634:MSWR:isotig11489	AT3G03780	0.0	*MS2*	methionine synthase 2	temp-0h	0.77
CUST_4912_PI429951308	reCj19509:MSWR:isotig19361	AT3G48560	0.0	*CSR1*	chlorsulfuron/imidazolinone resistant 1	temp-30d	0.85
CUST_8310_PI429951308	reCj22939:MSWR:isotig22791	AT2G36460	0.0	*FBA6*	Aldolase superfamily protein	temp-0h	0.86
CUST_8360_PI429951308	reCj22989:MSWR:isotig22841	AT1G66200	0.0	*GSR2*	hypothetical protein	temp-0h	0.89
CUST_6995_PI429951308	reCj21609:-SWR:isotig21461	AT2G29560	0.0	*ENOC*	cytosolic enolase	temp-0h	0.93

## Discussion

### Annual transcriptome dynamics vary between sites from autumn to spring but not in summer

Our 1-year period microarray data revealed dramatic changes in transcripts over the time course and varied expression patterns between the three sites (Fig 2). Interestingly, differences in the transcriptome dynamics were revealed from November to March. During the dormant period (from January to March), the transcriptome of Japanese cedar planted in the colder site (Yamagata) exhibited a higher PC2 score than the trees planted in the warmer sites (Ibaraki and Kumamoto) (Fig 2C). As annual ring growth of Japanese cedar is positively correlated with temperature in February and March [6], differences in the transcriptome during this season may reflect growth. The transition to the growth period (from March to April) began earlier at the transcriptome level in Kumamoto as compared with Yamagata and Ibaraki, and the transition to the dormant period (from November to December) occurred earlier in Yamagata than in Ibaraki and Kumamoto (Fig 2). This result agreed with the results of hierarchical clustering (Fig 3). The April samples from Kumamoto belonged to the same cluster as growth period samples, whereas the April samples in Yamagata and Ibaraki belonged to the same cluster as the dormant period samples. Also, the October samples from Yamagata and Ibaraki divided from the other growth period samples and created a sub-cluster. These transcriptome differences during the transition period in spring and autumn indicated that the growth period in Kumamoto was longer than that in Yamagata and Ibaraki; thus, the transcriptome differences could have contributed to the longer growth period in Kumamoto (Fig 1D). PCA of the microarray data also demonstrated continuous changes in the transcriptome throughout the dormant period, from November to March, at all three sites. Although Japanese cedar does not grow during the dormant period, these changes may reflect preparations for growth in the following spring.

Only small differences in the transcriptome were observed between the sites during the growth period. The transcriptome of all three sites exhibited higher PC1 scores from June to September, and the data plotted to a similar position (Fig 2B), even though the weekly mean air temperature on the sampling date at the three sites ranged from 19.3 to 27.6°C. Once the growth period began, the Japanese cedar transcriptome may not have been significantly influenced by temperature. In an analysis of Japanese cedar seedlings, Ujino-Ihara [34] reported that heat shock treatment (45°C, 3 h) induced approximately 3,000 differentially expressed unigenes. She also reported that heating pre-treatment reduced the number of differentially expressed genes induced. Thus, these data suggest that the transcriptome changes dramatically when the temperature exceeds a certain threshold and/or that Japanese cedar acclimatize to heat stress during the early stages of the active growth period under field conditions as the temperature gradually rises.

### Genes differentially expressed among the three sites

The comparison of ‘Yamagata vs. Kumamoto’ revealed the largest number of significant targets (1,473 targets) among the three comparisons (Fig 4), perhaps because these sites exhibited the largest difference in temperature throughout the year (Fig 1B). The comparisons of ‘Yamagata vs. Ibaraki’ and ‘Ibaraki vs. Kumamoto’ revealed 1,137 and 925 significant targets, respectively. The lower number of significant genes could be attributed to the intermediate temperature in Ibaraki.

The 75 common significant targets among the three comparisons may play an especially important role in environmental responses (Table 1). Among the 75 common targets, the expression of *eIF1A* showed the highest negative correlation (*r* = −0.95) with ‘temp-30d’. The mechanisms of translation initiation are fundamentally similar among all organisms, and two eukaryotic translation initiation factors are universally conserved, including eIF1A [35–37]. eIF1A is an important determinant of tolerance to NaCl stress in yeast and plants, and overexpression of *eIF1A* is associated with a slow-growth phenotype [37]. The higher expression of *eIF1A* in Japanese cedar at lower temperatures may be related to the response to cold stress and growth cassation at lower temperatures. A positive correlation (*r* = 0.77) was observed between long-term air temperature (‘temp-90d’) and expression of the gene encoding SAM-Mtase superfamily protein, which is a key enzyme in phenylpropanoid, flavonoid, and other metabolic pathways of biotechnological importance [38]. A negative correlation (*r* = −0.72) between ‘day length’ and *ABA1* expression was also identified (Table 1). The gene encoding highly ABA-induced PP2C protein 3 (*HAI3*) also exhibited a strong negative correlation with ‘day length’ (Table 6). Expression of ABA-related genes in accordance with changes in day length during xylem formation was also observed in the cambium region of Japanese cedar [39]. These data suggest that the expression of *ABA1* is involved in controlling endogenous ABA responses to day length and regulating the timing of xylem formation.

A total of 2,505 targets were differentially expressed in the three comparisons (Fig 4 and S4 Table). GO enrichment analysis indicated that the 2,505 differentially expressed targets included a significantly higher proportion of genes associated with the terms ‘response to stress’ and ‘response to abiotic stimulus’ (Table 3). These genes may play an important role in environmental adaptation. PLSR analysis indicated that the parameters of air temperature and day length subsequently or directly influenced the expression of most of the 2,505 common targets (Fig 5). To estimate the effects of the environment on the expression level of each target, we also calculated Pearson correlation coefficients, and 2,065 targets (82.4%) showed a strong correlation (r > 0.70) with the meteorological parameters examined in this study (Table 2). Air temperature–related parameters were correlated with 73.6% of the 2,505 differentially expressed targets, indicating that temperature is the dominant factor controlling the different transcriptomes between sites. This result was consisted with a study in rice [40, 41], which found that seasonal temperature changes are the dominant factor controlling transcriptome dynamics in the field. Interestingly, long-term air temperature (average air temperature over a 30-day period) was correlated with the expression of 17.2% of the 2,505 differentially expressed targets. These genes may be more affected by long-term temperature trends compared with short-term fluctuations in temperature, which could be more stable. This phenomenon indicates that Japanese cedar may be influenced by temperature trends over the previous 2 to 3 months.

The 441 targets that did not exhibit a strong correlation with meteorological parameters in this study may be influenced by other environmental parameters, such as soil conditions (e.g., temperature, moisture content, and nutrients), or the lack of correlation could perhaps be explained by examining other calculated parameters, such as cumulative temperature values as reported in an analysis of *FLC* gene expression in *Arabidopsis* [42]. Although we calculated the correlation to a single environmental parameter in this study, several environmental parameters may affect the expression level of a single target, as reported in rice [40]. Calculations of the effects of individual genes of interest could perhaps provide more details regarding the impact of environmental conditions on gene expression.

### Growth-related genes up-regulated as temperature increased

GO enrichment analysis indicated that the 824 targets positively correlated with parameters of air temperature included a significantly higher proportion of genes related to ‘microtubule cytoskeleton organization’, ‘cell wall organization or biogenesis’, and ‘growth’ (Table 5). Pfam enrichment analysis demonstrated a significantly higher proportion of targets including ‘microtubule binding’ domain, and all nine targets associated with ‘microtubule binding’ domain exhibited a strong positive correlation with the parameters of air temperature (Table 4 and S8 Table). These results indicate that Godai1 may promote formation of the microtubule cytoskeleton and growth in summer, especially in warmer sites. Nineteen targets corresponding to 17 *Arabidopsis* genes were related to ‘microtubule cytoskeleton organization’, including the *TON2* gene, which encodes a putative novel protein phosphatase 2A regulatory subunit essential for control of the cortical cytoskeleton in *Arabidopsis* [43]. Expression of *TON2* was positively correlated with ‘temp-60d’; thus, this gene may play an important role in controlling cytoskeleton-related genes. A total of 73 targets corresponding to 65 *Arabidopsis* genes were related to ‘growth’, including cellulose-related (cellulose synthase A catalytic subunit 1, cellulose synthase interactive 1, and cellulose synthase interactive 3) and expansin-related genes (expansin-A4). This phenomenon suggests that growth differences between sites could be influenced by the length of the growth period.

### Acclimation to harsh winter conditions at the colder site

Among the 2,505 differentially expressed targets in this study, 24 targets related to cold response exhibited a strong correlation with the parameters of air temperature, including *PRP31* and *FBP7* (S6 Table). *PRP31* is necessary for pre-mRNA splicing and regulation of the expression of cold-responsive genes [44], and *FBP7* is required for protein synthesis during temperature stress in *Arabidopsis* [45]. These genes may contribute to cold acclimation in winter, especially in colder regions. Our data also indicated the possibility of an important role for chitinases in over-wintering, especially in colder regions. Among the 2,505 differentially expressed targets, a sequence of six targets exhibited high homology to the genes encoding chitinase A, homolog of carrot EP3-3 chitinase, class V chitinase, and chitinase family protein. The expression of these genes exhibited a strong negative correlation with the parameters of air temperature (S4 Table). Plant chitinase has been implicated in defense responses against both biotic and abiotic stressors [46–49]. Diverse chitinases have been identified in spruce, and these enzymes act in concert to protect against freezing injury, store nitrogen, and induce growth cessation by promoting cell maturation [50].

Strong light in low temperature creates an imbalance between light energy absorption and energy use, leading to light stress in winter [51]. Eleven targets related to UV response exhibited a strong negative correlation with the parameters of air temperature, including *ATCSA-1*, *RUS1*, and *UVR7* (S7 Table). In *Arabidopsis*, *ATCSA-1* plays an important role in UV-B tolerance and genomic integrity [52], and *RUS1* and *UVR7* mutants are sensitive to UV light [53, 54]. These genes may contribute to photoprotection in winter, especially in colder areas. The photoprotective role of rhodoxanthin in Japanese cedar during cold acclimation is well known [55–58]. Several photoprotective processes that prevent severe damage during exposure to strong light in low-temperature conditions may function in Japanese cedar.

Stress-related hormones may play important roles in acclimating to harsh winter conditions, especially in colder areas. Among the 2,505 differentially expressed targets, five targets related to ABA and four targets related to ethylene showed a strong negative correlation to the parameter of air temperature or day length, indicating up-regulation of the expression of these targets in winter (Table 6). Both ABA and ethylene are known to play roles in regulating bud dormancy [59]. ABA is a major regulator of bud dormancy and promotes starch accumulation in the dormant phase by regulating the expression of starch- and sugar-related genes in grapevine (*Vitis vinifera* L.) buds [59, 60].

### Starch- and sugar-related genes exhibit varied annual expression patterns and differences between sites

Starch breakdown not only enhances cold and frost tolerance, it also contributes energy for bud breaking and shoot growth [61–66]. A previous report indicated that sugar concentration peaks in winter (January) in every part (upper, middle, and lower layer shoots and roots) of the seedlings of Japanese cedar [15]. Frost hardiness is also increased in winter, especially in colder sites [16]. Pfam enrichment analysis indicated a significantly higher proportion of targets associated with ‘Polysaccharide biosynthesis protein’ among the 2,505 differentially expressed targets (Table 4). In addition, 23 targets encoding starch- and sugar-related genes were differentially expressed between sites (Table 7). Among the starch degradation–related genes described in a previous report [32], the *BAM4* and *LSF1* genes were negatively correlated with ‘temp-0h’, and *GWD1* was negatively correlated with ‘day length’. These genes may promote starch degradation at lower temperatures in northern-latitude sites and may contribute to the difference in frost hardiness between sites. In contrast, three target genes related to starch biosynthesis [32] exhibited a positive correlation with parameters related to air temperature (*PGM*, *SS2*, and *APL2*), and these genes may contribute to starch biosynthesis in summer. The varied expression patterns and between-site differences in starch- and sugar-related genes may indicate that Godai1 biosynthesizes sugars and starches that are optimal for the environmental conditions.

### Lower temperature may induce energy accumulation in winter for growth in spring

In winter, especially in colder areas, Godai1 may prepare for growth in the upcoming spring by accumulating energy. Brassinosteroid, cytokinin, and auxin are well known as growth-related hormones. A total of eight targets related to these hormones showed a strong negative correlation with the parameters of air temperature in this study (Table 6). We also observed higher expression of genes related to fatty acid beta-oxidation and amino acid synthesis in lower temperatures, which could be related to generation of energy for growth. These results indicate the importance of low temperature to growth in the coming spring in a clone of Japanese cedar.

Beta-oxidation is the main pathway of fatty acid degradation [67]. GO enrichment analysis indicated that the 1,018 targets that were negatively correlated with parameters of air temperature included a significantly higher proportion of genes related to ‘fatty acid beta-oxidation’ (Table 5). This result appears to be consistent with those of previous reports of peanut (*Arachis hypogaea* L.) [68] and rice (*Oryza sativa* L.) [69], which exhibited up-regulation of genes related to fatty acid beta-oxidation under cold stress. Up-regulated expression of multiple beta-oxidation genes during germination was also reported in Tung tree (*Vernicia fordii*, Hemsl.) [70] and Upland cotton (*Gossypium hirsutum* L.) [71]. As fatty acid beta-oxidation generates energy for a growing embryo [71, 72], it may be an important mechanism to store lipids in winter as an energy source for growth in the coming spring, especially in colder regions.

Amino acids play multiple physiological roles in plants. For example, amino acids function as nitrogen carriers in transport systems, precursors for important metabolites, nitrogen storage molecules, stress response molecules, and signaling molecules [73]. Mori [17] reported that seedlings of Japanese cedar express approximately 20 amino acids, with citrulline, glutamate, and proline being the predominant amino acids in this species. Citrulline, a hydroxyl radical scavenger, accumulates in response to drought stress and nitrogen status in watermelon [74–76]. Mori [77] also reported that citrulline is a major compound involved in nitrogen translocation from roots to the shoot via the xylem in Japanese cedar. Glutamate plays a very important role in plant growth and development and in the response and adaptation to abiotic stress [78–80]. Proline plays a highly beneficial role in plants and also provides the cells sufficient energy to sustain rapid growth [81–83]. With respect to the high level of citrulline and low level of arginine accumulation, Japanese cedar may differ from other gymnosperm tree species that accumulate high levels of arginine [17, 84–86]. Seasonal changes in amino acids have been observed in coniferous trees [17, 87, 88]. In Japanese cedar, citrulline and proline accumulate during the active growth period and then decrease significantly, whereas glutamate exhibits less-marked seasonal changes [17]. In this study, the expression of genes involved in glutamate biosynthesis (*ALAAT2* and *ASP2*) [89], citrulline biosynthesis (*OCT* and peptidase M20/M25/M40 family protein), and proline biosynthesis (*P5CR*) were up-regulated as the temperature decreased (Table 8). The gene encoding peptidase M20/M25/M40 family protein demonstrated the highest negative correlation with 7-day mean air temperature among the six glutamate biosynthesis genes, regardless of season and site (Fig 6). This result indicated the importance of low temperature for amino acid biosynthesis. Interestingly, the expression patterns of amino acid biosynthesis genes in this study and the accumulation dynamics of amino acids reported by Mori [17] were not in accordance. The amino acid biosynthesis genes were up-regulated in winter when the temperature decreased, and the amino acids accumulated in the coming spring. It is known that some mRNAs are transcribed during cold acclimation and stored for the subsequent cold de-acclimation [90]. The up-regulation of amino acid biosynthesis–related genes at low temperature (during the dormant period) may represent preparation for growth in the coming spring. The reason for the higher expression of these genes in colder sites remains to be elucidated, however. Godai1 planted in colder regions may accumulate higher amounts of amino acids to promote primary growth in spring. Warmer winters due to climate change could reduce the expression of amino acid–related genes and affect growth in spring. Further study of amino acids in Japanese cedar could increase understanding of the adaptation mechanism during the transition from the dormant to active growth periods.

## Conclusions

We revealed changes in the annual transcriptome dynamics in a clone of Japanese cedar by analyzing rooted cuttings planted in three sites differing in terms of climate conditions. We identified a total of 2,505 differentially expressed targets among the three sites that may play important roles in enabling cuttings to adapt to local environmental conditions. The expression of 2,064 targets (82.4%) was affected by air temperature and day length, suggesting that these targets are directly or indirectly (subsequently) regulated by these environmental factors. Although short-term temperature was correlated with most of the genes, long-term temperature was also correlated with some genes, indicating that temperature trends may influence the expression of transcripts over several months. Targets that exhibited a strong negative correlation with air temperature included genes possibly related to cold tolerance and energy accumulation for the coming spring, such as amino acid biosynthesis, starch degradation, and fatty acid beta-oxidation genes. Transcriptome differences between sites observed from autumn to spring may reflect these differentially expressed targets. These results indicate the importance of adaptation to winter climate conditions in planting regions and the importance of low temperature to growth in the coming spring in a clone of Japanese cedar. Understanding how Japanese cedar adapt to various climate conditions is becoming more important due to changes in global climate in recent years. The results of this study may help elucidate the underlying biological mechanisms of environmental response in Japanese cedar.

Geographically, Japanese cedar natural forests range extensively from northern Honshu (30N, 130E) to Yakushima (40N, 140E) [91], and the genome-wide genetic diversity of natural populations and breeding core collections have been reported [92–96]. Tsumura et al. [93] studied the relationships between genotypes of natural populations and environmental variables and identified loci associated with the studied environmental variables. The results of the present study together with those of previous studies suggest the possibility of divergence of environmental responses in Japanese cedar. Although only the Godai1 clone was examined in the present study, other clones may show different environmental responses. Future studies examining additional clones selected from different regions could enhance our understanding of how Japanese cedar adapt to various climate conditions.

## Supporting information

S1 Fig Image of shoot sample, ‘A 10-cm portion of the lateral branch apex’. (TIF)

S2 Fig Expression patterns of five selected targets analyzed by microarray and qRT-PCR. Blue, green, and pink lines represent the average normalized intensity values in Yamagata, Ibaraki, and Kumamoto.
(TIF)

S3 Fig Annual tree height (A) and annual growth ratio (B) at the three sites.
(TIF)

S1 Table Sampling dates in Yamagata, Ibaraki, and Kumamoto. (XLSX)

S2 Table Temperature and day length parameters used in PLSR. *Sunlight data in Yamagata were obtained at the Yamagata observation site, and other meteorological data were obtained at the Higashine observation site.
(XLSX)

S3 Table Primers used in this study for qRT-PCR. (XLSX)

S4 Table The 2,505 significant differentially expressed targets identified by comparisons of the three sites. (XLSX)

S5 Table Three predictive components of PLS analysis. (XLSX)

S6 Table Targets related to the GO term ‘response to cold’ that exhibited a strong negative correlation with parameters of air temperature. (XLSX)

S7 Table Targets related to the GO term ‘response to UV’ that exhibited a strong negative correlation with parameters of air temperature. (XLSX)

S8 Table Targets enriched in the Pfam domain among the 2,505 differentially expressed targets. (XLSX)
