# Supplementary figures and images for: Changes in annual transcriptome dynamics of a clone of Japanese cedar (Cryptomeria japonica D. Don) planted under different climate conditions
Source: PLoS One. 2023 Feb 16;18(2):e0277797. doi: 10.1371/journal.pone.0277797 (PMC9934357; doi:10.1371/journal.pone.0277797)

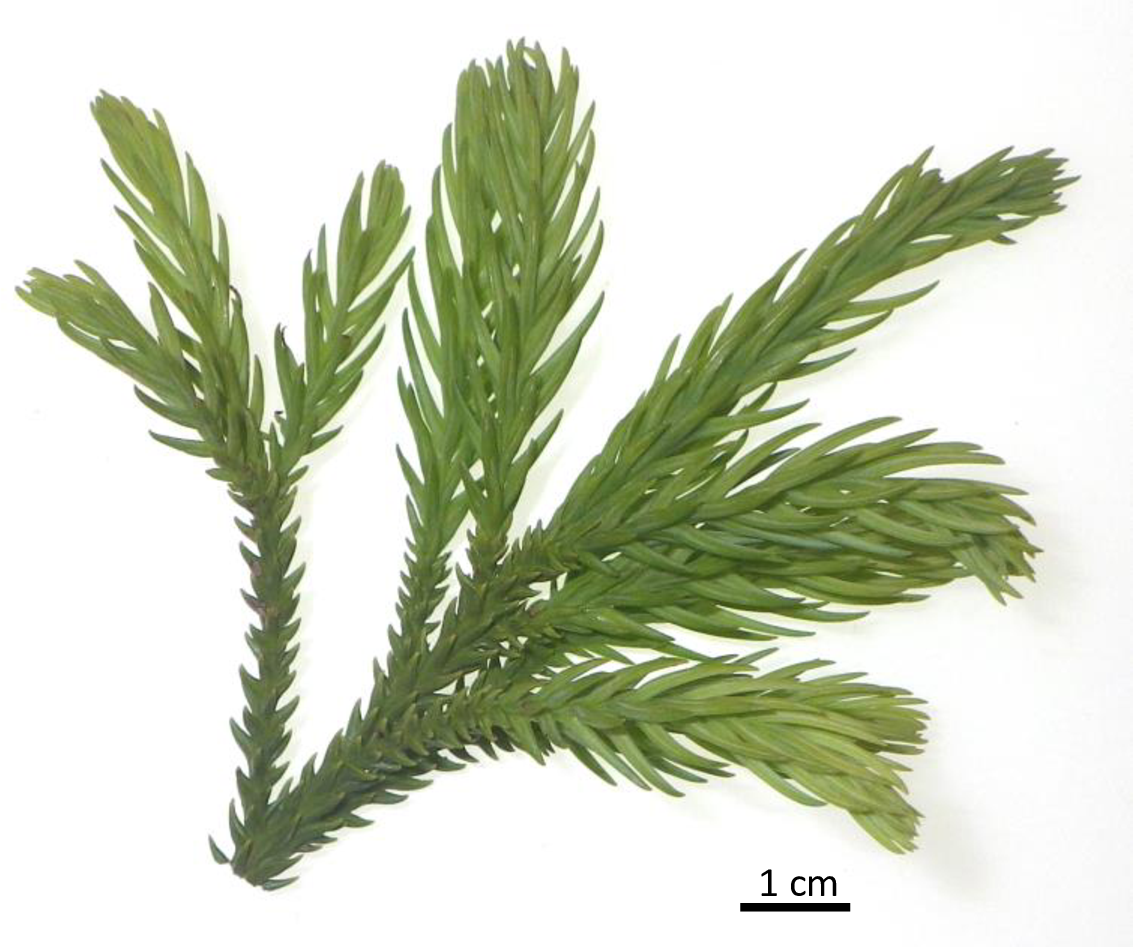

Supplement: S1 Fig — (TIF) [file pone.0277797.s001.tif]

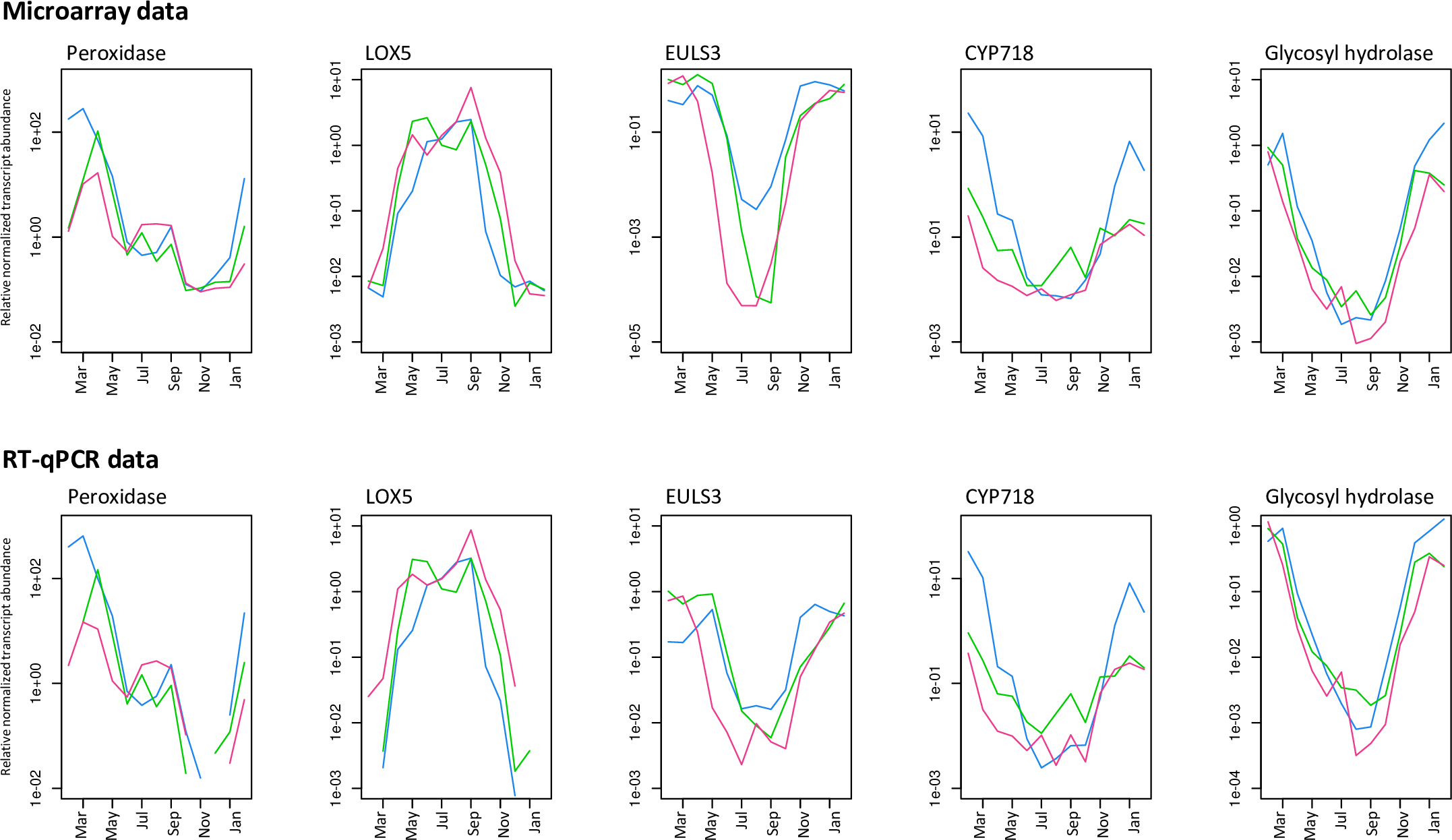

Supplement: S2 Fig — Blue, green, and pink lines represent the average normalized intensity values in Yamagata, Ibaraki, and Kumamoto. (TIF) [file pone.0277797.s002.tif]

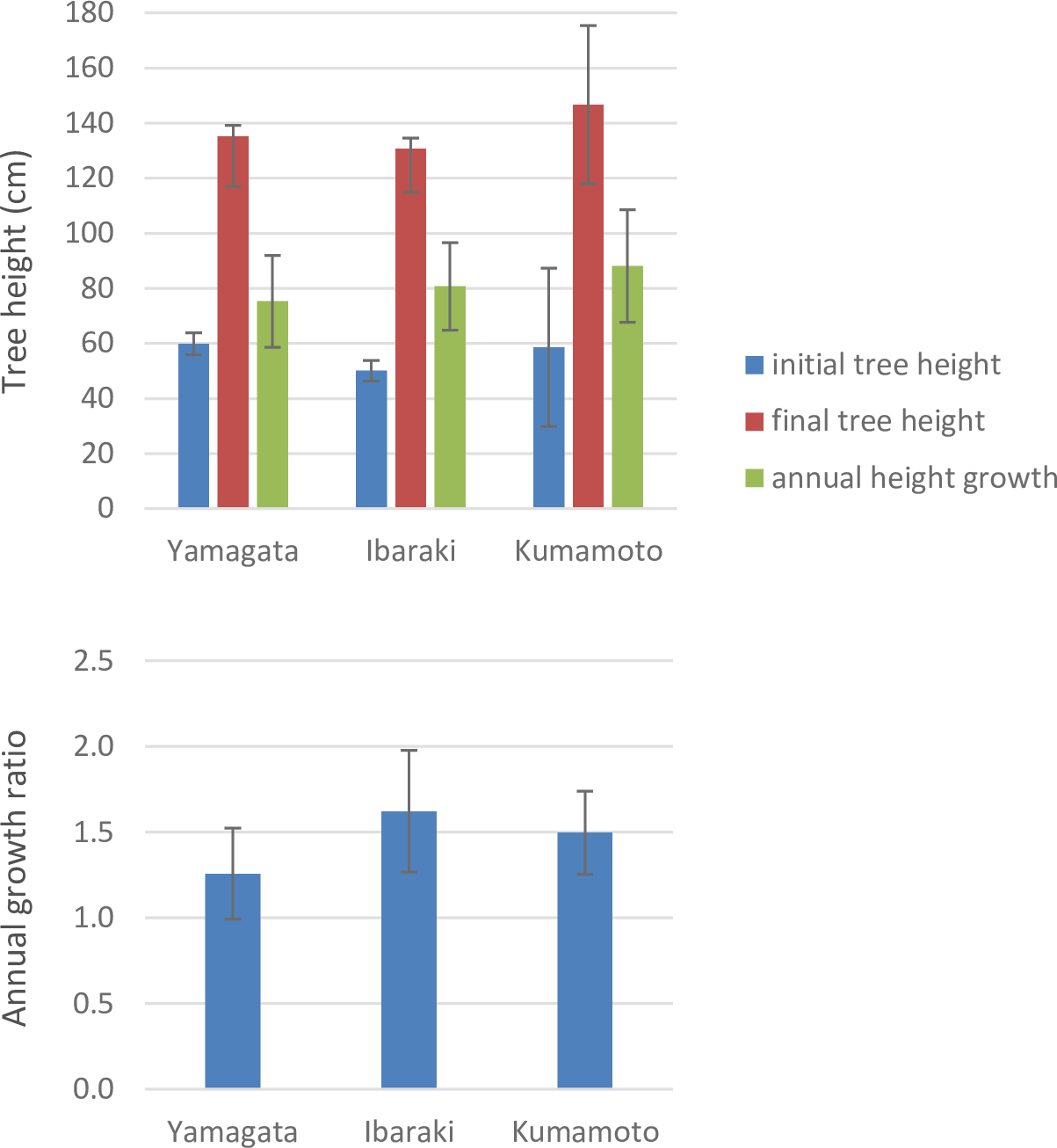

Supplement: S3 Fig — Annual tree height (A) and annual growth ratio (B) at the three sites. (TIF) [file pone.0277797.s003.tif]
